# Phase Stability of High Entropy (Mg,Ni,Co,Cu,Zn)O from Temperature‐Resolved Synchrotron Diffraction: Tetragonal Distortion and Guggenite Phase

**DOI:** 10.1002/smll.202406634

**Published:** 2024-12-19

**Authors:** Mauro Coduri, Martina Fracchia, Stefano Checchia, Maela Manzoli, Catherine Dejoie, Paolo Ghigna, Umberto Anselmi‐Tamburini

**Affiliations:** ^1^ Chemistry Department University of Pavia via Taramelli 16 Pavia 27100 Italy; ^2^ INSTM National Inter‐University Consortium for Materials Science and Technology Via G. Giusti 9 Florence 50121 Italy; ^3^ ESRF – The European Synchrotron 71, avenue des Martyrs Grenoble 38000 France; ^4^ Department of Drug Science and Technology and NIS – Centre for Nanostructured Interfaces and Surfaces Via P. Giuria 9 Turin 10125 Italy

**Keywords:** guggenite, high entropy oxides, in situ XRPD

## Abstract

The temperature‐resolved structure evolution of quinary and quaternary equimolar oxides containing Mg, Ni, Zn, Co, and Cu is investigated by in situ synchrotron diffraction. Important structural modifications occur already at mild temperatures and depend on the elements involved. All quaternary compounds with χ(Cu) = 0.25 within 250–500 °C show a tetragonal phase. The fraction of Cu dictates the degree of the tetragonal distortion: for χ(Cu) = 0.20 only local distortions are observed, which do not occur in the absence of Cu. Further heating restores the original cubic phase, followed by the segregation of other phases, which depend on the elements available. In the presence of both Cu and Mg, the segregation of guggenite is observed, a phase with a cation connectivity similar to the cubic high entropy oxide, but with a suitable coordination environment for Cu atoms by allowing strong elongation of octahedra. Guggenite acts as a buffer layer facilitating the gradual diffusion of Cu^2+^ from the cubic high entropy oxide to tenorite. To conclude, it is demonstrated for the first time that the demixing of the prototypal high entropy oxide does not involve just tenorite or binary oxides, it is rather mediated by the formation of guggenite.

## Graphical abstract

Quinary and quaternary high entropy oxides of the (Mg,Ni,Co,Cu,Zn)O family show complex phase and structural evolution upon heating, which depends on the elements involved. At mild temperatures they undergo a tetragonal distortion, whose distortion is defined by the fraction of copper. When both Cu and Mg are present, they segregate as guggenite (Cu_2_MgO_3_‐like) before forming tenorite (CuO).

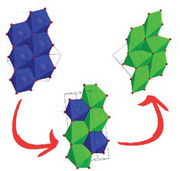

## 1 Introduction

High‐entropy oxides (HEOs) are an emerging class of ceramics in which a specific crystal structure can be stabilized due to the presence of a large number of cations, typically five or more.^[^ ^1^, ^2^ ^]^ This concept was directly extended from high‐entropy alloys, where the “cocktail effect” and configurational disorder are usually exploited to obtain novel materials with peculiar properties.^[^ ^3^, ^4^, ^5^ ^]^ The first HEO with composition Ni_0.2_Co_0.2_Mg_0.2_Zn_0.2_Cu_0.2_O (hereafter *HEO‐5*), reported in 2015, crystallizes with a rock‐salt (RS) structure, space group *Fm‐*3*m*.^[^ ^1^ ^]^ Nowadays, HEOs are produced in many different crystal structures,^[^ ^6^ ^]^ the most common being spinels,^[^ ^7^, ^8^, ^9^, ^10^ ^]^ perovskites,^[^ ^11^, ^12^ ^]^ and fluorites,^[^ ^13^, ^14^ ^]^ though in principle any crystal structure can accommodate a HEO.^[^ ^15^, ^16^, ^17^, ^18^ ^]^ HEOs have already shown to be promising for several applications, especially in the field of catalysis^[^ ^19^, ^20^, ^21^, ^22^, ^23^, ^24^ ^]^ or energy production and storage.^[^ ^25^, ^26^, ^27^, ^28^, ^29^, ^30^, ^31^, ^32^ ^]^

Besides searching for new compositions with different crystal structures, there is currently a significant effort focused on investigating the prototype *HEO‐5* to determine: i) the compositional range of stability,^[^ ^33^ ^]^ ii) the possible insertion of foreign cations,^[^ ^34^, ^35^, ^36^ ^]^ and iii) the factors controlling phase stability and structural distortions, especially with regard to the configurational entropy and the nature of the components. In this respect, three of the components of *HEO‐5* (MgO, NiO and CoO) exhibit the same RS crystal structure as *HEO‐5*, while CuO and ZnO crystallize at room temperature as tenorite (space group *C*2/*c*) and as wurtzite (space group *P*6_3_ *mc*), respectively. An enthalpic cost is then required to force Cu^2+^ and Zn^2+^ into the regular octahedral environment of the RS structure. This is generally believed to be compensated by the configurational entropy, arising from the large number of components in equimolar composition. However, the effective role of the configurational entropy, especially in view of the solubility equilibria of the oxides, still needs to be fully addressed.^[^ ^37^, ^38^, ^39^ ^]^ In this respect, we have recently demonstrated that the formation of a single high entropy phase cannot be inferred only on the basis of the configurational entropy, but it rather depends on the chemical nature of the components.^[^ ^33^, ^38^ ^]^ Many fundamental and structural aspects regarding the stability of the high entropy RS phase and its possible structural distortions remain open questions. For instance, by increasing the molar fraction of copper from 0.20 to 0.28 while keeping the other four cations equimolar, the compound deviates from the ideal cubic structure.^[^ ^40^ ^]^ A similar phenomenon, evident in X‐ray diffraction (XRD) patterns as a selective broadening of all the reflections with the exception of those related to the *hhh* family, was observed in the prototypical compound after post‐annealing treatments at 250–300 °C.^[^ ^20^, ^40^ ^]^ In both cases, the origin of such phenomena was attributed to the Cu^2+^ strong Jahn‐Teller (JT) effect,^[^ ^40^, ^41^, ^42^ ^]^ i.e., the tendency of the Cu^2+^ ions to distort their coordination from a regular octahedral environment, which instead is the case of a RS crystal structure. For low Cu content, the Cu^2+^ ions are diluted: they do not interact with each other and induce only some local disorder.^[^ ^41^ ^]^ For larger Cu content, the JT distortion promotes a transformation into a tetragonal phase.^[^ ^40^ ^]^ Evidences of a tetragonal distortion were observed also in quaternary oxides.^[^ ^43^ ^]^ Non‐cooperative JT distortion in *HEO‐5* at room temperature was also demonstrated by X‐ray absorption spectroscopy (XAS)^[^ ^1^, ^44^ ^]^ and confirmed by DFT calculations.^[^ ^45^ ^]^ The stability of *HEO‐5* as a function of temperature is generally investigated by collecting XRD data at room temperature (RT) after quenching, either monitoring the progress of the reaction from the single component to the *HEO‐5*,^[^ ^9^, ^46^, ^47^ ^]^ or by post‐annealing after the single phase has formed.^[^ ^1^, ^44^ ^]^ Hong et al.^[^ ^47^ ^]^ revealed that, in the formation of *HEO‐5*, the first step of the reaction occurs between MgO and CoO to give a mixed RS phase, to which NiO adds at higher temperatures. Increasing the temperature favors the dissolution of ZnO into RS, finally followed by CuO. Once a single RS phase has formed, treatments between ≈650 °C and ≈850 °C lead to a multiphase system, involving the segregation of spinel and tenorite phases, depending on experimental conditions,^[^ ^1^, ^44^ ^]^ while at higher temperatures the single RS phase is again stabilized.

All the above studies suggest a complex scenario occurring in *HEO‐5* over a wide range of temperatures. However, structural studies are available only at some selected temperatures, which vary for each investigation. In view of the many possible applications of HEOs, which span over a wide temperature range, knowing the phase evolution during the thermal treatment can provide valuable information for understanding the mechanisms at work, the identification of the phases involved and their chemical composition. To address this gap, we present an in situ XRD investigation monitoring the structural evolution in the 25–900 °C temperature range of the quinary *HEO‐5* and of all the quaternary oxides, obtained by selectively removing one of the cations. Quaternary oxides are labeled as “no” followed by the element symbol of the missing cation: *noMg* (Ni_0.25_Co_0.25_Cu_0.25_Zn_0.25_O), *noNi* (Mg_0.25_Co_0.25_Cu_0.25_Zn_0.25_O), *noCo* (Mg_0.25_Ni_0.25_Cu_0.25_Zn_0.25_O), *noCu* (Mg_0.2__5_Ni_0.25_Co_0.25_Zn_0.25_O), and *noZn* (Mg_0.25_Ni_0.25_Co_0.25_Cu_0.25_O). The comparison of these different HEOs allowed to highlight the role played by each cation and the consequence of its excess compared to the reference quinary *HEO‐5*. We evidenced the main role of Cu^2+^ in the quinary oxide, which, upon heating, first drives a distortion of the ideal cubic phase and finally segregates into a tenorite phase. We demonstrate for the first time that, before complete segregation, the diffusion of Cu^2+^ ions from RS to tenorite occurs via the formation of an intermediate guggenite (Cu_2_MgO_3_‐like) phase, rather than simply demixing into the single binary oxides. Guggenite is a Cu‐rich oxide of the Cu_2_BO_3_ family, with B being a divalent cation, with intermediate structural characteristics between RS and tenorite, i.e., with cations distributed between a regular and a distorted octahedral site.^[^ ^48^ ^]^ In the framework of the system (Ni, Mg, Co, Cu, Zn)O, guggenite has been identified so far only with B = Mg,^[^ ^48^, ^49^, ^50^ ^]^ Co,^[^ ^51^ ^]^ and in the ternary system (Ni, Mg, Cu)O.^[^ ^52^ ^]^ In addition, it was observed for B = Ca^[^ ^53^ ^]^ and Sr^[^ ^54^ ^]^ and very recently in a high entropy system in the presence of Mn.^[^ ^55^ ^]^ The compound Cu_2_MgO_3_ is very recently attracting attention in composites in the field of catalysis and energy conversion.^[^ ^56^, ^57^ ^]^ In addition to the role of guggenite, we also show that a fully tetragonal phase, a distortion of the original RS phase, occurs by combining mild thermal treatment (250–400 °C) in the quaternary oxides in the excess of Cu (χ(Cu) = 0.25).

## 2 Results

### 2.1 Room Temperature Structure and Phase Composition

**Figure 1:**
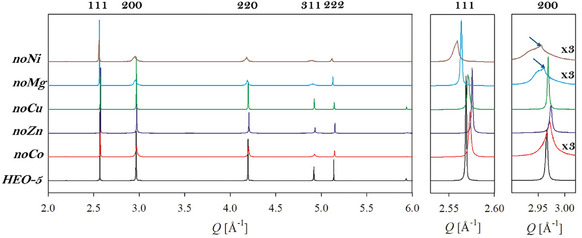
High resolution XRPD patterns collected at RT. The insets on the right highlight the profile of the first two diffraction peaks. The arrows point to the signal evidence of residual cubic phase.

The detailed RT structure and phase composition of *HEO‐5* and quaternary oxides were first investigated by high‐resolution X‐ray powder diffraction (HR‐XRPD) using synchrotron radiation. The very high brilliance of the source allows to spot impurities with very low concentration and to accurately describe the line profiles. This is particularly relevant in the field of high entropy materials, to probe slight phase inhomogeneities otherwise undetectable using laboratory instruments.^[^ ^58^, ^59^ ^]^ The HR‐XRPD patterns collected at RT are displayed in **Figure** **1**.

All samples are single‐phase except for small traces of spinel, guggenite, and Pt, the latter originating from the crucible used for synthesis. Impurities never exceed 1.5% wt. However, while the quinary compound exhibits sharp peaks with no sign of phase separation or peak splitting, the quaternary oxides display selective peak broadenings, as shown in the inset of Figure 1, where the 111 and 200 reflections are magnified. Indeed, while the full width at half maximum (FWHM) of the 111 is comparable to that of standard Si (0.003 deg. in these experimental conditions^[^ ^60^ ^]^), the 200 reflection broadens significantly, even by a factor of 10 (see **Table** **1**) in comparison with the quinary HEO. Such a selective *h*00 broadening was attributed to the local JT distortion produced by Cu^2+[^ ^40^ ^]^ and is particularly evident when the molar fraction of copper is 0.25 or higher.

**Table 1:** Structural parameters computed by Rietveld refinements and single peak fitting.

	*HEO‐5*	*noCo*	*noZn*	*noCu*	*noMg*	*noNi*
frac. Cubic	1	1	1	1	0.01	0.03
*a* / Å	4.236373(7)	4.22969(3)	4.22598(3)	4.23341(1)	4.2435(1)	4.2517(1)
*adp*(M)	0.00687(5)	0.00523(8)	0.0062(1)	0.00362(6)	0.0037(1)	0.0057(2)
*adp*(O)	0.0123(5)	0.0127(3)	0.0132(5)	0.0049(2)	0.0125(3)	0.0092(4)
FWHM(111)	0.00436(2)	0.00959(3)	0.00488(2)	0.00951(3)	0.006529(3)	0.0218(6)
FWHM(200)	0.0146(4)	0.0502(4)	0.0165(3)	0.0108(2)	0.11*	0.18*
FWHM (200)/(111)	3.35	5.24	3.37	1.14	17.46*	8.25*
frac. Tetragonal	0	0	0	0	0.99	0.97
*a*_T_ / Å	–	–	–	–	3.00850(5)	3.0161(1)
*c*_T_ / Å	–	–	–	–	4.2253(1)	4.2334(4)
*c*_T_/*a*_T,N_	–	–	–	–	0.993	0.992

^*^FWHM extracted without peak fitting because of overlapped reflections.

The reasons behind this selective broadening need further clarification. The broadening of a diffraction peak reflects the finite structural coherence in the direction perpendicular to the corresponding plane. This can be associated to a finite crystal size, to microstrain, or any other kind of defects,^[^ ^61^ ^]^ such as stacking fault^[^ ^62^ ^]^ or antiphase domain,^[^ ^63^ ^]^ that limit the structural ordering in a given direction. It must be noted that these specimens were produced via solid‐state reaction, using long high‐temperature treatments, ruling out the possibility of small crystallite size. In addition, similarly to,^[^ ^43^ ^]^ the largest peak broadening is accompanied, in the case of *noMg* and *noNi*, by peak asymmetries, suggesting the transformation into a lower symmetry system. As already noted by Bérardan et al., the occurrence of a rhombohedral distortion can be excluded, since it would show an opposite behavior than the one observed here, with the splitting of *hhh* instead of *h*00 reflections. The patterns are rather consistent with a tetragonal distortion, which indeed leads to the splitting of all reflections, but the *hhh*. More details about the tetragonal phase will be discussed later in this manuscript. A possible monoclinic distortion was proposed by Usharani et al.,^[^ ^64^ ^]^ but such a transformation would induce the splitting of all reflections, *hhh* included. In conclusion, only *noMg* and *noNi* show a tetragonal distortion at RT, while all other specimens are cubic, although characterized by strong structural disorder. The peak broadening, evidence of a lack of structural coherence, is prominent only for those reflections related to the cubic‐to‐tetragonal transformation. This indication suggests that in the absence of peak splitting a tetragonal distortion might still occur, but it is limited at the local scale. This can be pictured as a domain structure where the tetragonal phase only exists as a short‐range order and has, therefore, limited coherence length. Thus, in the following, we will refer to a tetragonal phase only in the presence of peak splitting or asymmetries that can be clearly related to a phase transition to a tetragonal system. Otherwise, when the reflections sensitive to the tetragonal distortion are symmetrically broadened, we considered the tetragonal distortion only as a local effect, and the system is deemed as cubic. Taking this into account, we will consider *noNi* and *noMg* samples as tetragonal. A small contribution of a cubic, well‐ordered RS phase, characterized by sharp peaks, is still observed in *noNi* and *noMg* (see arrows in the inset of Figure 1), corresponding to 1% and 3%, respectively, from Rietveld refinements. A scheme of the cubic‐to‐tetragonal distortion is displayed in Figure S1 (Supporting Information). Normalizing the lattice parameter *a*_T_ of the tetragonal phase to the cubic setting according to $a_{T,n}=a_{T}\;\sqrt{2}$, the magnitude of the long‐range tetragonal distortion is estimated as the ratio of the (normalized) tetragonal lattice parameters *c_T_*/*a*_*T*,*n*_. For a cubic system, the ratio is 1. For *noNi* and *noMg* at RT the ratio is ≈0.99, suggesting a moderate, yet significant, tetragonal distortion. The lattice parameters are tabulated in Table 1, together with other structural information. Another useful information extracted from Rietveld refinement is the atomic displacement parameter (*adp*). In the case of ionic compounds, rather than the actual amplitude of vibrations, *adp*s provide information on the site disorder. Indeed, the larger the site disorder, the faster the angular decay of the diffracted intensity, and the larger the *adp*.^[^ ^65^, ^66^ ^]^ Table 1 shows that *noCu* has by far the lowest values of *adp* among the samples with RS structure. Since the lowest *adp*s are observed in the absence of Cu and *adp*s are a fingerprint of structural disorder, it follows that most of the structural disorder is induced by Cu. The cation *adp*s of tetragonal specimens are as low as that of *noCu*, because the static disorder of the cations is partially accounted for by the lowering of symmetry.

**Figure 2:**
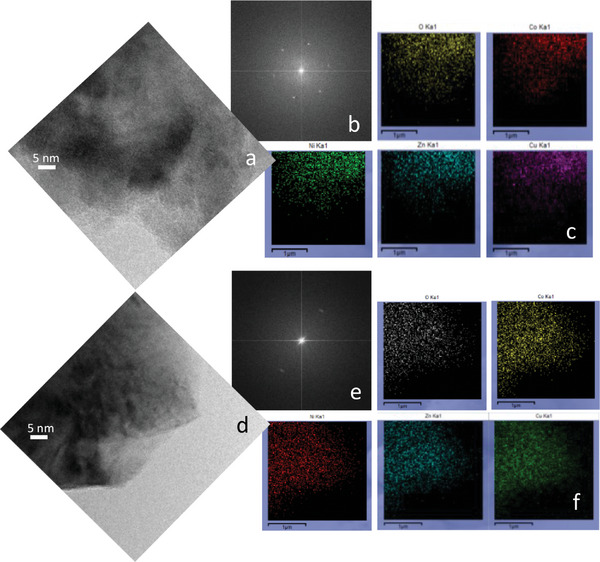
a,d) HR‐TEM representative images of *noMg*. b,e) Fast Fourier Transform of the images in a and d, respectively. c,f) EDS maps of the regions shown in a and d for all the cations and oxygen. Instrumental magnification: 400000x.

An extended high‐resolution transmission electron microscopy (HR‐TEM) combined with EDS characterization was carried out on *HEO‐5* (Figure S2, Supporting Information) and on all the quaternary oxides (**Figure** **2**; Figures S3–S6, Supporting Information) to investigate the tetragonal distortion by checking crystallinity and chemical homogeneity of the materials.

First of all, it is worth noting that no modifications of sample morphology and composition were observed upon exposition under the electron beam. Overall, the results of the HR‐TEM characterization carried out on all samples are in good agreement with the information obtained by HR‐XRPD performed at RT and shown in Figure 1. Indeed, the HR‐TEM analysis of the *HEO‐5* sample revealed a uniform distribution of the elements (Figure S2b, Supporting Information) and a cubic phase, as indicated by the presence of the spot related to the (200) planes in the Fast Fourier Transform (FFT) of the image shown in panel *c*. Conversely, the removal of Mg gave rise to strong local tetragonal distortion. This is evident by comparing the spacings measured in the FFTs of two different regions of the sample and reported in Figure 2, in which representative HR‐TEM images of *noMg* are reported along with the corresponding FFTs and the EDS maps. Analogously, the tetragonal distortion was observed also for the *noNi* specimen (Figure S3, Supporting Information). Conversely, *noCo*, *noCu* and *noZn* showed the typical spacing of the RS cubic structure (Figures S4–S6, Supporting Information). The chemical homogeneity was assessed also in these cases, without any evident element enrichment in different regions of the samples.

### 2.2 Temperature Evolution of Structure and Phase Composition

#### 2.2.1 HEO‐5

**Figure 3:**
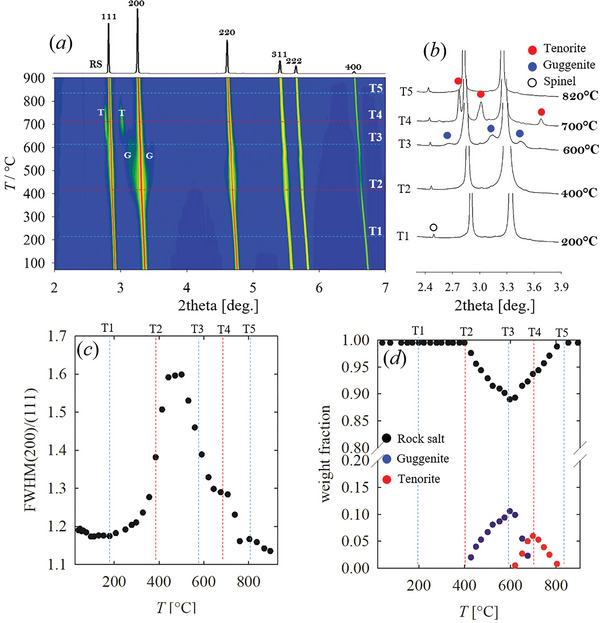
a) Temperature‐resolved XRPD patterns of *HEO‐5* reported in Log scale, with the highest temperature pattern (900 °C) plotted in linear scale on the top, indexed according to a RS structure. T stands for tenorite, G for guggenite. b) Low angle portion of XRD patterns cut at 1.5 k counts. d) FWHM(200)/FWHM(111) as a function of temperature. e) Weighted phase fractions computed by Rietveld refinements.

**Figure 4:**
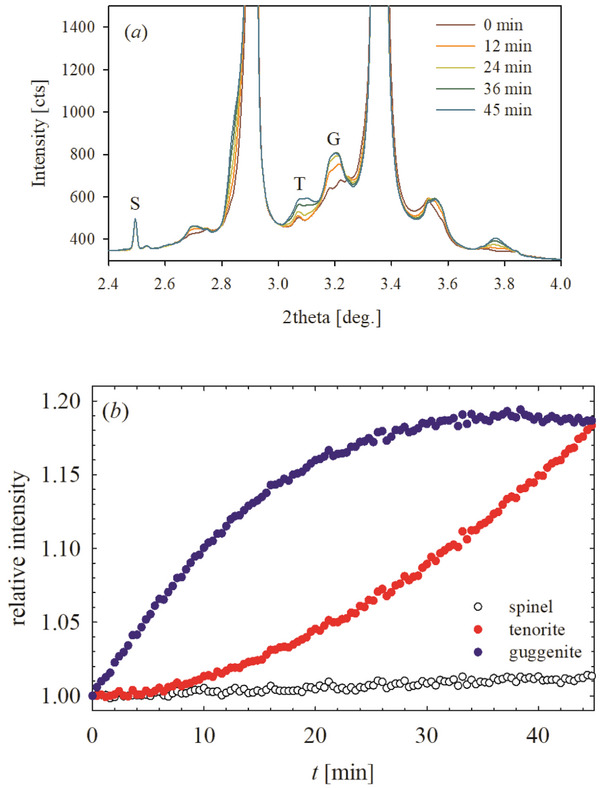
a) low intensity portion of XRPD patterns collected as a function of time while holding T at 600 °C for 45 min. b) Intensity evolution for spinel, tenorite and guggenite recorded at points S (spinel), T (tenorite), and G (guggenite) shown in part (a) chosen as a reference of the above phases. The intensities are normalized against the values recorded when starting the holding.

An overview of the temperature evolution of the XRPD patterns for *HEO‐5* is given in **Figure** **3**a. The 2D map (*a*) reports the peaks intensity for the main RS phase on a logarithmic scale for temperatures up to 900 °C. The pattern at the highest temperature is reported on the top of the panel. The high brilliance of the synchrotron source evidence a rich set of events in the low‐intensity region, that would be passed unnoticed using a laboratory diffractometer. Figure 3b highlights the low‐intensity areas of some selected patterns. Up to ≈200 °C, no significant variation occurs. Further heating, however, produces an increase in the broadening of the 200 and related reflections, which show a significant dependence on temperature. This is evidenced in Figure 3c, where the ratio of the FWHM of the 200 over the 111 reflections is reported as a function of temperature. The ratio is nearly constant up to 200 °C (T_1_ in Figure 3), and then starts increasing up to ≈400 °C (T_2_), where it reaches a maximum. Further heating up to 600 °C (T_3_) produces a narrowing in the RS peak and the appearance of broad, low‐intensity reflections, which can be attributed to a guggenite phase. Guggenite peaks are broad and become only slightly sharper at higher temperature (see below). The evolution of the phase composition with temperature, as determined by Rietveld refinements, is given in Figure 3d. The fraction of guggenite increases up to ≈10% wt., reaching the maximum at ≈600 °C. The narrowing of RS peaks slows down above 600 °C, where tenorite (CuO) starts to form at the expense of guggenite. The largest fraction of tenorite (≈7%) is observed at ≈700 °C (T_4_). Further heating consumes the tenorite phase, leading again to a pure RS specimen with reflections, 200 included, even sharper than at RT. No evidence of wurtzite or spinel, except for the small traces already detected at RT, was observed. To gain insight into the kinetic of formation of guggenite, we heated a *HEO‐5* specimen at 600 °C, the temperature at which the largest fraction of guggenite was observed (see Figure 3d), and we followed the phase evolution as a function of time. The XRD patterns are shown in **Figure** **4**a, while the intensity evolution of the fraction of spinel, guggenite and tenorite phases are given in panel (*b*). The intensity of the reference reflection of the spinel phase impurity is roughly constant, while that of guggenite increases as soon as the temperature is reached and stabilizes after ≈30 min. On the other hand, the tenorite intensity keeps increasing.

#### 2.2.2 noMg

**Figure 5:**
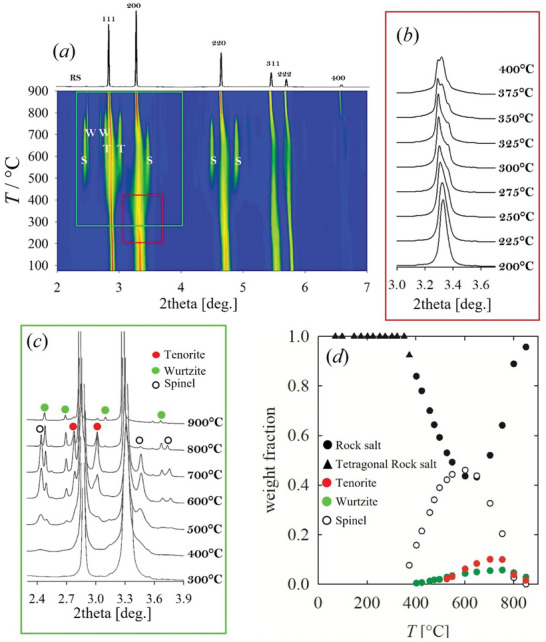
a) Temperature‐resolved XRPD patterns of noMg reported in Log scale, with the highest temperature pattern (900 °C) plotted in linear scale on the top, indexed according to a RS structure; T stands for tenorite, G for guggenite, S for spinel, W for wurtzite. b) Temperature evolution of the 200 reflection in RS setting from 200 to 400 °C, consistent with the red area in panel (a). c) Low‐intensity scale highlighting the formation and evolution of secondary phases from 300 to 900 °C, consistent with the green area in panel (a). d) Weighted phase fractions computed by Rietveld refinements.

Let us now consider the quaternary compositions, starting from *noMg*. The removal of Mg from *HEO‐5* induces a tetragonal distortion that is already evident at RT, resulting in a significant peak broadening and only ≈1% of residual cubic phase. The temperature evolution of the XRD patterns is shown in **Figure** **5**a,b. In contrast to *HEO‐5*, above ≈400 °C the specimen decomposes into several phases. In addition, the RS phase undergoes a significant distortion. Above ≈200 °C, the 200 reflection becomes progressively asymmetric, a behavior observed for all reflections except for the *hhh*. At 300 °C, the 200 is fully split (*c*/*a* = 0.984), with an uneven intensity distribution between the two components (110 and 002 in the tetragonal setting). No superstructure peaks are observed, although we cannot exclude they might be too broad or not intense enough to be detected. Superstructure peaks would arise, as an example, in case of a cooperative JT distortion,^[^ ^67^ ^]^ where O ions would displace from the special position leading to different metal‐oxygen bond lengths. Having no evidence of such a distortion, we modelled the pattern in space group *I*4/*mmm*, as proposed in,^[^ ^20^, ^40^ ^]^ with cations sharing the 2a site (0,0,0) and O in 2b (0,0,1/2). An example of Rietveld refinement of a tetragonal specimen is shown in Figure S7 (Supporting Information). Further heating progressively reduces the amount of the tetragonal phase to form cubic RS, as evidenced by the reappearance of the cubic 200 in between the tetragonal 110 and 002 reflections, demonstrating the coexistence, in this temperature range, of well‐defined cubic and tetragonal phases (see Figure 5b). At higher temperatures the RS phase becomes unstable, leading to the segregation of spinel, wurtzite, and later tenorite. These phases are consistent with the single binary oxides constituting the HEO, i.e., Co_3_O_4_, ZnO, and CuO, respectively. This occurs only after the tetragonal distortion has vanished. At 400 °C traces of spinel begin to appear, accompanied by the formation, to a lower extent, of wurtzite. The relative amounts of these two phases increase rapidly with temperature, especially for the spinel, which at 600 °C becomes the major phase. Tenorite is first observed at ≈550 °C, a temperature much lower than in the quinary oxide. Above 600 °C the reduction in the amount of the spinel phase is accompanied by the growth of tenorite, which exceeds wurtzite and reaches its maximum amount at ≈750 °C. At higher temperatures even this oxide is consumed like the others. From ≈800 °C the RS phase is fully restored, with peaks narrower than at RT and with no evidence of tetragonal distortion (Figure 5a–c). It is then evident that *HEO‐5* and *noMg* show equivalent structure at ≈800 °C, despite the significant differences at RT (see Figure 1).

#### 2.2.3 noCu

**Figure 6:**
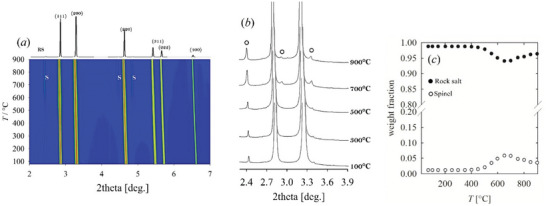
a) Temperature‐resolved XRPD patterns of noCu in Log scale. b) Low angle portion of the patterns cut at 1.5 k counts highlighting spinel peaks. c) Weighted phase fractions computed by Rietveld refinements.

At RT, the *noCu* solid solution presents the smallest FWHM (200)/(111) among the investigated compositions. The high‐temperature evolution is shown in **Figure** **6**, highlighting again the low‐intensity region (*a*‐*b*). The 2D plot reveals the appearance of a small amount of spinel occurring from ≈500 °C, leading to ≈5% spinel phase, which is partly consumed to reform RS above ≈700 °C. (*c*) Residual 3% spinel phase is retained at 900 °C. The full‐scale patterns show no evidence of tetragonal distortion, not even in terms of broadening of the 200 RS reflection.

#### 2.2.4 noCo, noZn, and noNi

The temperature‐resolved patterns of *noCo*, *noZn*, and *noNi* are reported in Figures S8–S10 (Supporting Information). As observed for *noMg*, though at a higher temperature, the distorted tetragonal phase is consumed to form a cubic RS phase. However, above 400 °C, guggenite rather than the binary oxides is observed, thus suggesting that the presence of Mg is necessary for the formation of this phase. The maximum concentration of guggenite is reached above 500 °C and is larger for *noCo*. Further heating induces its consumption to form tenorite. Traces of wurtzite are observed above 800 °C in *noCo*, but they are readily consumed to form RS. No spinel is formed at high temperatures. The impurities observed at RT (Pt in *noZn* and spinel in *noCo*) remain unchanged, except for thermal expansion, throughout the temperature cycle.

## 3 Discussion

A comparison of the temperature behavior of quinary and quaternary compounds can be used to determine the role played by each cation in the stabilization of the RS phase. To this purpose, we can compare the temperature evolution of i) peak broadening ii) tetragonal distortion iii) cell parameters and iv) nature and fraction of the segregated phases.

### 3.1 Broadening

Figure S11 (Supporting Information) compares the broadening of the 200 reflection of RS for *HEO‐5* and *noCu*. In the quaternary compounds, the 200 peak is much broader than the other reflections, and the splitting owing to the tetragonal distortion makes the estimation of the FWHM less reliable (see Experimental Section). Full plots of FWHM are given in Figure S12 (Supporting Information). When the cubic RS phase is restored at high temperatures, the peaks are much sharper than at RT. Whereas the plots in Figures S11 and S12 (Supporting Information) refer to the 200 reflection, all reflections, including the 111, broaden in the same temperature range, even though to a different extent. This suggests the formation of a non‐homogeneous system, where the distribution of cell parameters related to RS phases with slightly different elemental compositions acts as an extra contribution to peak broadening. The only specimen that does not display such a broadening upon heating is the one free of copper (*noCu*). The other quaternary compounds show a significant broadening already at RT, which leads to a peak splitting at ≈300–400 °C. In *HEO‐5*, on the contrary, the splitting of the 200 is not observed. This indicates that the molar fraction of Cu is the main factor controlling the distortion. Molar fractions equal to 0.25 in the quaternary compounds, 0.20 in *HEO‐5* and 0 in *noCu*, induce, respectively, peak splitting, only broadening and no effect at all. Another aspect to consider is the kinetics of the process. For χ(Cu) = 0.20, no transformation toward a tetragonal system is observed. However, here we are monitoring the structure while heating at a constant rate (2 °C min^−1^), and we cannot exclude that a tetragonal transformation might be observed allowing the system to equilibrate at a given temperature. For this reason, we monitored the structural evolution of *HEO‐5* during isothermal treatment for 45 min at 400 °C and 500 °C, corresponding to the temperatures where the maximum peak broadening is observed upon heating. Examples of patterns and the evolution of FWHM during the isotherms are shown in Figure S13 (Supporting Information). Except for a progressive symmetric peak broadening, no evidence of tetragonal phase is observed. At 500 °C the broadening of the 200 reflection reaches a plateau, without any evidence of splitting. This evidences that the *HEO‐5* composition does not undergo a transition into a tetragonal system even at intermediate temperatures.

### 3.2 Tetragonal Distortion and Lattice Parameters

The tetragonal phase has been observed in all quaternary compounds except for *noCu*. The estimation of the relative amount of cubic and tetragonal phases is unreliable because of the peak broadening, except for the limited temperature range where the maximum distortion is reached, where cubic and tetragonal signals can be resolved. Therefore, it is more reliable to monitor the transformation by following the extent of the tetragonal distortion than its relative amount.

**Figure 7:**
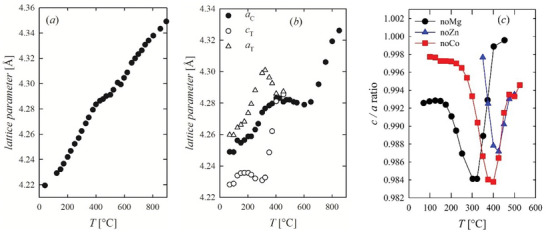
Cubic (full) and tetragonal (empty) RS lattice parameters as a function of temperature for *a*) *HEO‐5* and b) *noMg*; c) tetragonal distortion observed for *noMg*, *noZn*, and *noCo*.

**Figure** **7**a,b reports the cell parameters as a function of temperature of the RS phase for *HEO‐5* and *noMg* compositions. The tetragonal cell parameters show a different thermal expansion; indeed, *a*_T_ expands steeply while *c*_T_ shrinks. This trend continues until the maximum difference between *a*_T_ and *c*_T_ is achieved. Further heating narrows the gap between the two tetragonal cell parameters and restores the cubic phase. A similar trend is observed for the other quaternary oxides, as reported in Figure S14 (Supporting Information). The magnitude of the tetragonal distortion is plotted in Figure 7c. In this context, *noZn* and *noCo* can be considered cubic at RT, while *noMg* and *noNi* are tetragonal. The temperature corresponding to the maximum distortion of *noMg* is observed at ≈300 °C, and it increases to ≈350 °C for *noNi* and to ≈400 °C for *noCo* and *noZn*. The Rietveld refinement of the pattern corresponding to the maximum distortion of *noMg*, performed in space group *I*4/*mmm*, provides a satisfactory fit, as shown in Figure S16 (Supporting Information). Besides the tetragonal distortion, the evolution of the cell parameters upon temperature provides interesting information about the distribution of the elements between the different phases. Indeed, the occurrence of a particular phase does not imply that its composition resembles the one of the corresponding binary oxide, or, oppositely, a distribution of all available cations. Details are discussed in the Supporting Information. *HEO‐5* displays linear thermal expansion until ≈500 °C, where the expansion slows down for ≈100 °C (Figure 7a). This effect is observed at the same temperature interval where guggenite segregates. Guggenite is a Cu‐ rich phase, and its formation depletes the Cu^2+^ fraction in the RS phase. Let us now consider *noMg*. In this case, a tetragonal distortion is observed at the lowest temperatures. At ≈400 °C, the cubic RS phase is restored, but its unit cell contracts between ≈400 and ≈600 °C. In this same temperature range, *noMg* demixes into RS, spinel, and wurtzite. Considering that Zn segregates as wurtzite, due to the low solubility of the other cations in this phase, and Co^2+^ is likely in the spinel phase, the residual RS is expected to be rich in Ni^2+^, which is the cation with the smallest ionic radius in octahedral coordination (Table S2, Supporting Information). As in this case, neither tenorite nor guggenite is observed in this temperature range; the phase most likely to host Cu^2+^ is the spinel. See Text S2 (Supporting Information) for further discussions. A break in the thermal expansion is also observed for *noCo* and *noZn*, again concomitant to the segregation of guggenite and the removal of Cu from the RS phase.

**Figure 8:**
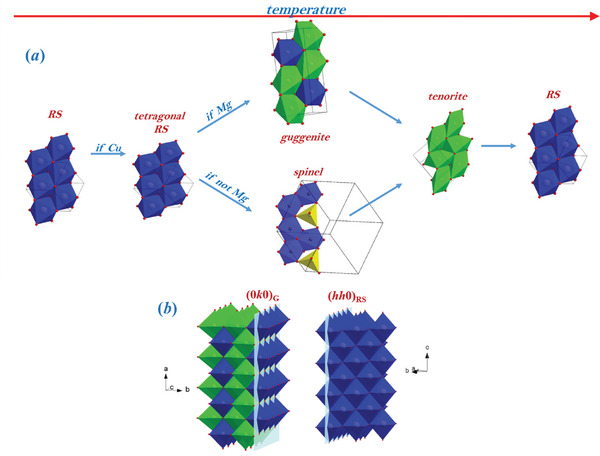
a) Sketch of the phases supposed to host Cu upon heating. Blue octahedra indicate regular (or nearly regular) octahedra, consistent with a random cation distribution, green octahedra are preferentially occupied by Cu. Tetrahedra in spinel phase are in yellow. The top part of the figure highlights the correlation between RS and guggenite cell parameters. b) Matching a (0*k*0) regular guggenite layer with a (*hh*0) RS layer.

**Table** **2** summarizes the temperature of the formation, maximum concentration and vanishing of the secondary phases. The step common to all the solid solutions containing Cu is the segregation of tenorite, which starts above 600 °C and is consumed between 800 and 900 °C. The specimen *noMg* is an exception, as tenorite starts to form already at 500 °C and is consumed at lower temperatures than in the other quaternary compounds. A similar behavior applies to the tetragonal phase. At RT, *noMg* is nearly fully tetragonal. Heating increases the distortion, which reaches a maximum at a lower temperature than the other quaternary oxides. Similar considerations apply to other secondary phases. Whereas the critical temperatures of the transformations seem to be affected by the extent of the distortion retained at RT, the nature of the phases formed depends on the available cations. It is clear that the selective broadening and splitting of RS peaks is triggered by the tendency of Cu^2+^ to impose a JT distorted local coordination, leading to a tetragonal order, either locally (in *HEO‐5*) or long‐range (in the quaternary compounds). Only in the absence of Mg, the removal of Cu follows a different route, involving the formation of a spinel phase. In the other cases, the JT distortion promotes the formation of guggenite. Whatever the phase formed, from ≈550–600 °C it is consumed to form tenorite. This is sketched in **Figure** **8**, where similar portions of the different crystallographic structures are compared to highlight similarities and differences among the phases under investigation.

**Table 2:** Temperature (in °C) corresponding to the formation, maximum fraction (bold), and vanishing of secondary phases. G stands for guggenite, S for spinel, T for tenorite, and W for wirtzite.

	*HEO‐5*	*noCu*	*noMg*	*noCo*	*NoZn*
Tetragonal	–	–	RT **325** 425	225 **400** 600	300 **425** 500
G	400 **600** 700	–	–	375 **550** 700	400 **550** 650
S	–	400 **650** *	375 **600** 800	–	–
T	625 **700** 800	–	500 **750** 825	625 **750** 900	575 **675** 900
W	–	–	400 **750** 900	750 **825** 875	–

### 3.3 Guggenite

A point to be clarified is the mechanism that leads to the formation of guggenite, rather than spinel, in the transition from the RS to the tenorite phase. As shown in Figure 4, the analysis of XRD patterns recorded while equilibrating *HEO‐5* at 600 °C revealed that the fraction of guggenite increased steeply until reaching a plateau, while the fraction of tenorite kept on growing. This behavior suggests that guggenite represents an intermediate phase, whose growth is rapid at the beginning and then is inhibited by its decomposition to form tenorite. In this regard, it must be noted that in the binary system MgO‐CuO, where guggenite Cu_2_MgO_3_ is reported to be a stable phase,^[^ ^50^ ^]^ the guggenite forms as an intermediate layer between RS (MgO) and tenorite, as also discussed in the Supporting Information. Considering the relative rate of formation of the two phases at 600 °C (Figure 4b), we speculate that guggenite nucleates first, then part of Cu^2+^ ions of guggenite starts forming the tenorite, which grows maintaining constant the amount of guggenite. This is consistent with the blurred peak observed for guggenite, even 200 °C above its nucleation temperature (see, e.g., Figure 5). This is also probably the reason why guggenite has been so far overlooked as a secondary phase in HEOs. Guggenite XRD signals are very broad and low in intensity, and they can be spotted on a laboratory diffractometer only when the fraction of guggenite is large, which happens at ≈500–600 °C, a temperature range that is generally not considered, as most of the attention is paid to the higher temperature range. The above mechanism implies a structural similarity between RS and guggenite and between guggenite and tenorite. RS consists of a fcc arrangement of O ions with cations filling octahedral holes. All octahedra are regular and identical. Those sketched in Figure 8 for the RS phase are lying within the (111)_RS_ plane. The cations, ideally, are randomly distributed on the same site. The tetragonal distortion involves the elongation of *a* and the contraction of *c*. The experimental XRD patterns are consistent with the *I*4/*mmm* space group with the cations and the O ions occupying special positions. Excluding possible cooperative JT distortions, such a configuration leads to an overall contraction of the CuO_6_ octahedra, which is the opposite of the elongation generally observed in conventional JT distortion involving Cu^2+^.^[^ ^68^ ^]^ Rak et al.^[^ ^45^ ^]^ proposed the formation of randomly oriented elongated octahedra, together with a small fraction of strongly contracted octahedra, which globally induce the contraction of the *c* cell parameter. It appears that the elongation of a Cu‐O pair within an octahedron is accommodated by the contraction of the other Cu─O pair along the same direction. This octahedra distribution recalls the structural motif of guggenite. Guggenite crystallizes in orthorhombic space group *Pmmn* with lattice parameters *a*_G_ ≈4.0 Å, *b*_G_ ≈9.3 Å, *c*_G_ ≈3.2 Å. The guggenite unit cell can be expressed as a function of that of the RS phase (*a*_RS_), as shown in Figure S18 (Supporting Information), where the undistorted RS unit cell is sketched with orange lines. All metal‐oxygen pairs along *a*_G_ share the same interatomic distance, which corresponds roughly to half of the original RS unit cell. Owing to the different centering of the unit cell, the other guggenite cell parameters are related to the base diagonal of the RS: *c*_G_ corresponds to half of it, while the long axis *b*_G_ to one and a half. Details are given in Figure S18 (Supporting Information). Guggenite maintains the same octahedra connectivity as RS, with the cations distributing among two different sites: one nearly regular, (2a, blue in Figure 8) which fits a bivalent cation and one elongated (4e, green in Figure 8), suitable for the JT distortion of Cu^2+^. According to the structure reported in^[^ ^48^ ^]^ for Cu_2_MgO_3_, the distorted site has 4 equatorial interatomic distances at ≈2.0 Å, and two axial distances at 2.5–2.7 Å, while the more regular site has 4 equatorial distances at ≈2.15 Å and two axial distances at 2.0 Å, thus showing a compressed octahedron, consistent with the local picture proposed by Rak et al.^[^ ^45^ ^]^ The different cation site multiplicity imposes a double fraction of distorted octahedra with respect to the undistorted ones, leading to ≈66% of cations being Cu^2+^. It turns out that the structure of guggenite and RS are closely related. In this respect, the structures in Figure 8 are displayed to highlight the connectivity between the same octahedra: when no octahedron is distorted (blue), i.e., in the absence of Cu^2+^, the atomic structure is the same of RS and *a*_G_ matches *a*_RS_. When a large fraction of Cu^2+^ ions are available, they tend to order with the guggenite structure, giving rise to a mixture of regular (blue) and distorted (green) octahedra. The ordering of the distorted octahedra triggers the transition from distorted RS to guggenite. Similarly, one can conceive the transformation from guggenite to tenorite. Tenorite is again composed of octahedra, with the same connectivity as in RS and guggenite. However, in tenorite, all octahedra are extremely elongated forming layers with octahedra oriented in different directions. The distortion is further increased with respect to guggenite, thus leading to a monoclinic system, space group *C*2/*c*. Guggenite combines the structural characteristics of RS and tenorite. This is evident by looking at the stacking along the *b* axis of guggenite (see Figure 8b), where layers composed of only regular or only irregular octahedra alternate with a 1:2 ratio. Thus, guggenite can be viewed as the stacking of RS‐ and tenorite‐like structural units. Moreover, the layer composed of regular octahedra corresponds to the (*hh*0) plane of RS (Figure 8b), thus creating a favorite path for the RS to guggenite transformation. Such a picture holds in the presence of both Mg and Cu. In the absence of Mg, the guggenite phase does not form upon heating. In such event, Cu^2+^ ions are hosted within the spinel phase, together with Co^3+^ ions formed as consequence of the oxidation of Co^2+^ to Co^3+^ at intermediate temperatures. In the spinel, Cu^2+^ finds another environment allowing to maintain its octahedral coordination, which has to be to energetically more favored compared to the RS phase. To conclude, the present study provided a detailed picture of the phase evolution of the Mg_0.2_Ni_0.2_Co_0.2_Zn_0.2_Cu_0.2_O high entropy oxide over temperature, showing that important structural modifications occur already in a temperature range of interest for many technological applications (e.g., catalysis). We demonstrate the active role of the guggenite phase, generally observed between 400 and 600 °C, which assists the demixing process, led by copper, of *HEO‐5* into different components. The presence of guggenite, as well as the distortion into a tetragonal phase, should be taken into account when predicting the behavior of Cu‐containing high entropy oxides.

## 4 Conclusion

The high temperature behavior of quinary and quaternary compounds of equimolar oxides containing Mg, Ni, Zn, Co, and Cu was investigated. The specimens were produced by solid‐state reaction leading to pure RS phases and homogenous cation distribution. Room temperature high‐resolution powder diffraction revealed that *noMg* and *noNi* specimens showed tetragonal symmetry, while the quinary and the other quaternary HEOs are cubic, even though important line profile effects suggest a disordered local environment, likely arising from JT distortion triggered by Cu^2+^ cations. The heating of all quaternary compounds containing Cu^2+^ induces a clear, long‐range tetragonal distortion, more evident than at RT. The splitting of RS diffraction peaks, evidence of a tetragonal distortion, is observed only when the molar fraction of Cu^2+^ is above 0.20; only a broadening is observed at a content of 0.20 (*HEO‐5*) and neither broadening nor splitting is noticed in the absence of Cu. Where present, the tetragonal phase transforms back to cubic RS at temperatures higher than 650 °C and the transition is followed by the segregation of other phases, the nature of which depends on the elements available. In the presence of Cu^2+^ and Mg^2+^, this phenomenon is accompanied by the segregation of guggenite, which has a similar cation connectivity to RS, but makes a more suitable coordination environment for Cu atoms by allowing strong elongation of octahedra. Accordingly, it was found that the residual RS phase present in this temperature range has a much lower fraction of Cu. It should be noted that guggenite forms even when the transformation into a tetragonal phase is not achieved, i.e., in the quinary oxide. Guggenite acts as an intermediate phase facilitating the transition from RS to tenorite and is found to be stabilized by the Mg^2+^ ions. When Mg^2+^ is missing, the high entropy oxide demixes, forming spinel and wurtzite, the former becoming the dominant phase at higher temperatures. At ≈600 °C (500 °C for *noMg*), guggenite (or spinel) reaches its maximum concentration and it is then consumed to form tenorite. Further heating allows the dissolution of Cu into the RS, which, at such a high temperature, presents no evidence of tetragonal distortion, not even at the local scale. To conclude, the present study provided a detailed picture of the phase evolution of the prototypical high entropy oxide over temperature, showing that important structural modifications occur already at mild temperatures. It was demonstrated for the first time that the demixing of HEO does not involve just tenorite or the other single oxides, but it is rather mediated by the formation of guggenite in the range between 400–600 °C. Moreover, significant deviations from the ideal cubic structure occur already at 300 °C. The emergence of either fully tetragonal or guggenite‐like high entropy phases offers new possibilities in the panorama of existing high‐entropy oxides, allowing for future exploration of new structures and compositions.

## 5 Experimental Section

The specimens were prepared through conventional solid‐state reaction starting from the commercial oxides (MgO, ZnO, CuO, NiO, CoO; Aldrich, purity > 99%). The oxides were mixed in due stoichiometry, pressed uniaxially into pellets, fired at 1200 °C for 6 h, and air‐quenched. Since Cu_2_O impurities were detected, due to CuO reduction at the high temperatures in air, a further thermal treatment at 1000 °C for 15 h followed by air quenching was added to guarantee phase purity. The *noCu* specimen was treated at a higher temperature (1350 °C) to complete the reaction. The second step at 1000 °C was not performed as Cu was missing in this sample and no secondary phases were detected from the laboratory diffractometer. The structural investigation at RT was carried out by combining HR‐XRPD, scanning electron microscopy (SEM), and HR‐TEM. Room‐temperature HR‐XRPD patterns were collected at the ID22 beamline of the European Synchrotron Radiation Facility (ESRF, Grenoble, France) at an incident wavelength of λ_1_ = 0.3545 Å (≈35 keV), using the setup equipped with crystal analyzers.^[^ ^60^, ^69^ ^]^ HR‐TEM images were obtained by using a side entry Jeol 3010‐UHR microscope operating at 300 kV, with a LaB_6_ filament and equipped with an Oxford Inca Energy TEM 300 EDS X‐ray analyzer by a Link ISIS 200 detector. Digital micrographs were collected with an Ultrascan 1000 camera and processed with a Gatan digital micrograph. Before the experiments, the samples, in the form of powders, were deposited on a gold grid covered with a lacey carbon film. High‐temperature XRD patterns were collected at the ID15A beamline of the ESRF, at an incident wavelength of λ_2_ = 0.1240 Å (≈100 keV), using a Pilatus 2 m CdTe detector (Dectris) placed 1100 mm away from the specimen.^[^ ^70^ ^]^ The powders were packed into quartz capillaries (Hilgenberg) with 0.5 mm diameter and rotated during the acquisition to improve the statistical orientation of the grains. Heating was provided by using a hot‐air blower (Cyberstar) placed at 5 mm from the specimen. Thermal calibration was performed using a corundum standard. XRD data were collected during constant heating at 2 °C min^−1^. Due to instrumental issues, patterns of the *noNi* specimen were collected only at a few selected temperatures up to 750 °C. Further isotherms on a fresh *HEO‐5* specimen were performed at 400, 500, and 600 °C. Rietveld refinements were performed using the GSAS‐II package.^[^ ^71^ ^]^ Single‐peak fitting to extract peak position, intensity and FWHM was performed using WinPLOTR.^[^ ^72^ ^]^ In the case of overlapping peaks, the FWHM had been estimated by measuring the angular range between the head and the tail of the peak taken at half of the highest intensity point.

## Conflict of Interest

The authors declare no conflict of interest.

## Supporting information



Supporting Information

## Data Availability

The data that support the findings of this study are available from the corresponding author upon reasonable request.

## References

[1] C. M. Rost , E. Sachet , T. Borman , A. Moballegh , E. C. Dickey , D. Hou , J. L. Jones , S. Curtarolo , J.‐P. Maria , Nat. Commun. 2015, 6, 8485. 26415623 10.1038/ncomms9485 PMC4598836

[2] C. Oses , C. Toher , S. Curtarolo , Nat. Rev. Mater. 2020, 5, 295.

[3] E. P. George , D. Raabe , R. O. Ritchie , Nat. Rev. Mater. 2019, 4, 515.

[4] M.‐H. Tsai , J.‐W. Yeh , Mater. Res. Lett. 2014, 2, 107.

[5] Y. Zhang , T. T. Zuo , Z. Tang , M. C. Gao , K. A. Dahmen , P. K. Liaw , Z. P. Lu , Prog. Mater. Sci. 2014, 61, 1.

[6] M. Fracchia , M. Coduri , P. Ghigna , U. Anselmi‐Tamburini , J. Eur. Ceram. Soc. 2024, 44, 585.

[7] J. Dąbrowa , M. Stygar , A. Mikuła , A. Knapik , K. Mroczka , W. Tejchman , M. Danielewski , M. Martin , Mater. Lett. 2018, 216, 32.

[8] M. Fracchia , M. Manzoli , U. Anselmi‐Tamburini , P. Ghigna , Scr. Mater. 2020, 188, 26.

[9] A. Mao , H.‐X. Xie , H.‐Z. Xiang , Z.‐G. Zhang , H. Zhang , S. Ran , J. Magn. Magn. Mater. 2020, 503, 166594.

[10] M. Coduri , M. Fracchia , M. Guerrini , C. Dejoie , P. Ghigna , U. A. Tamburini , J. Eur. Ceram. Soc. 2023, 43, 2728.

[11] S. Jiang , T. Hu , J. Gild , N. Zhou , J. Nie , M. Qin , T. Harrington , K. Vecchio , J. Luo , Scr. Mater. 2018, 142, 116.

[12] D. A. Vinnik , E. A. Trofimov , V. E. Zhivulin , S. A. Gudkova , O. V. Zaitseva , D. A. Zherebtsov , A. Y. Starikov , D. P. Sherstyuk , A. A. Amirov , A. V. Kalgin , S. V. Trukhanov , F. V. Podgornov , Nanomaterials 2020, 10, 268. 32033483 10.3390/nano10020268 PMC7075219

[13] J. Gild , M. Samiee , J. L. Braun , T. Harrington , H. Vega , P. E. Hopkins , K. Vecchio , J. Luo , J. Eur. Ceram. Soc. 2018, 38, 3578.

[14] L. Spiridigliozzi , C. Ferone , R. Cioffi , G. Dell'Agli , Acta Mater. 2021, 202, 181.

[15] Z. Teng , L. Zhu , Y. Tan , S. Zeng , Y. Xia , Y. Wang , H. Zhang , J. Eur. Ceram. Soc. 2020, 40, 1639.

[16] D. A. Vinnik , E. A. Trofimov , V. E. Zhivulin , O. V. Zaitseva , S. A. Gudkova , A. Y. Starikov , D. A. Zherebtsov , A. A. Kirsanova , M. Häßner , R. Niewa , Ceram. Int. 2019, 45, 12942.

[17] F. Ding , C. Zhao , D. Xiao , X. Rong , H. Wang , Y. Li , Y. Yang , Y. Lu , Y.‐S. Hu , J. Am. Chem. Soc. 2022, 144, 8286. 35472274 10.1021/jacs.2c02353

[18] Y. Sun , H. Xiang , F.‐Z. Dai , X. Wang , Y. Xing , X. Zhao , Y. Zhou , J. Adv. Ceram. 2021, 10, 596.

[19] S. H. Albedwawi , A. AlJaberi , G. N. Haidemenopoulos , K. Polychronopoulou , Mater. Des. 2021, 202, 109534.

[20] M. Fracchia , P. Ghigna , T. Pozzi , U. Anselmi Tamburini , V. Colombo , L. Braglia , P. Torelli , J. Phys. Chem. Lett. 2020, 11, 3589. 32309955 10.1021/acs.jpclett.0c00602 PMC8007101

[21] H. Xu , Z. Zhang , J. Liu , C.‐L. Do‐Thanh , H. Chen , S. Xu , Q. Lin , Y. Jiao , J. Wang , Y. Wang , Y. Chen , S. Dai , Nat. Commun. 2020, 11, 3908. 32764539 10.1038/s41467-020-17738-9 PMC7413391

[22] H. Chen , J. Fu , P. Zhang , H. Peng , C. W. Abney , K. Jie , X. Liu , M. Chi , S. Dai , J. Mater. Chem. A 2018, 6, 11129.

[23] F. Tavani , M. Fracchia , A. Tofoni , L. Braglia , A. Jouve , S. Morandi , M. Manzoli , P. Torelli , P. Ghigna , P. D'Angelo , Phys. Chem. Chem. Phys. 2021, 23, 26575. 34812450 10.1039/d1cp03946f

[24] Y. Pan , J.‐X. Liu , T.‐Z. Tu , W. Wang , G.‐J. Zhang , Chem. Eng. J. 2023, 451, 138659.

[25] A. Sarkar , L. Velasco , D. Wang , Q. Wang , G. Talasila , L. de Biasi , C. Kübel , T. Brezesinski , S. S. Bhattacharya , H. Hahn , B. Breitung , Nat. Commun. 2018, 9, 3400. 30143625 10.1038/s41467-018-05774-5 PMC6109100

[26] N. Qiu , H. Chen , Z. Yang , S. Sun , Y. Wang , Y. Cui , J. Alloys Compd. 2019, 777, 767.

[27] P. Ghigna , L. Airoldi , M. Fracchia , D. Callegari , U. Anselmi‐Tamburini , P. D'Angelo , N. Pianta , R. Ruffo , G. Cibin , D. O. de Souza , E. Quartarone , ACS Appl. Mater. Interfaces 2020, 12, 50344. 33124794 10.1021/acsami.0c13161 PMC8016163

[28] C. Zhao , F. Ding , Y. Lu , L. Chen , Y.‐S. Hu , Angew. Chem., Int. Ed. 2020, 59, 264. 10.1002/anie.201912171 31621145

[29] D. Wang , S. Jiang , C. Duan , J. Mao , Y. Dong , K. Dong , Z. Wang , S. Luo , Y. Liu , X. Qi , J. Alloys Compd. 2020, 844, 156158.

[30] D. Wang , Z. Liu , S. Du , Y. Zhang , H. Li , Z. Xiao , W. Chen , R. Chen , Y. Wang , Y. Zou , S. Wang , J. Mater. Chem. A 2019, 7, 24211.

[31] D. Callegari , M. Coduri , M. Fracchia , P. Ghigna , L. Braglia , U. A. Tamburini , E. Quartarone , J. Mater. Chem. C 2022, 10, 8994.

[32] S. Schweidler , M. Botros , F. Strauss , Q. Wang , Y. Ma , L. Velasco , G. Cadilha Marques , A. Sarkar , C. Kübel , H. Hahn , J. Aghassi‐Hagmann , T. Brezesinski , B. Breitung , Nat. Rev. Mater. 2024, 9, 266.

[33] M. Coduri , L. R. Magnaghi , M. Fracchia , R. Biesuz , U. Anselmi‐Tamburini , Chem. Mater. 2024, 36, 720.

[34] D. Hong , Y.‐H. Choi , J. Alloys Compd. 2024, 985, 174029.

[35] D. Bérardan , S. Franger , D. Dragoe , A. K. Meena , N. Dragoe , Phys. Status Solidi (RRL) – Rapid Res. Lett. 2016, 10, 328.

[36] M. P. Jimenez‐Segura , T. Takayama , D. Bérardan , A. Hoser , M. Reehuis , H. Takagi , N. Dragoe , Appl. Phys. Lett. 2019, 114, 122401.

[37] S. J. McCormack , A. Navrotsky , Acta Mater. 2021, 202, 1.

[38] M. Fracchia , M. Coduri , M. Manzoli , P. Ghigna , U. A. Tamburini , Nat. Commun. 2022, 13, 2977. 35624095 10.1038/s41467-022-30674-0 PMC9142508

[39] S. S. Aamlid , M. Oudah , J. Rottler , A. M. Hallas , J. Am. Chem. Soc. 2023, 145, 5991. 36881986 10.1021/jacs.2c11608

[40] D. Berardan , A. K. Meena , S. Franger , C. Herrero , N. Dragoe , J. Alloys Compd. 2017, 704, 693.

[41] G. Anand , A. P. Wynn , C. M. Handley , C. L. Freeman , Acta Mater. 2018, 146, 119.

[42] C. M. Rost , Z. Rak , D. W. Brenner , J.‐P. Maria , J. Am. Ceram. Soc. 2017, 100, 2732.

[43] W. Mnasri , D. Bérardan , S. Tusseau‐Nenez , T. Gacoin , I. Maurin , N. Dragoe , J. Mater. Chem. C 2021, 9, 15121.

[44] A. D. Dupuy , I.‐T. Chiu , P. Shafer , E. Arenholz , Y. Takamura , J. M. Schoenung , J. Eur. Ceram. Soc. 2021, 41, 6660.

[45] Z. S. Rák , J.‐P. Maria , D. W. Brenner , Mater. Lett. 2018, 217, 300.

[46] J. Sushil , A. Kumar , A. Gautam , M. I. Ahmad , Mater. Chem. Phys. 2021, 259, 124014.

[47] W. Hong , F. Chen , Q. Shen , Y. Han , W. G. Fahrenholtz , L. Zhang , J. Am. Ceram. Soc. 2018, 102, 2228.

[48] M. Winkelmann , H. A. Graf , B. Wagner , A. W. Hewat , Z. Kristallogr. – Cryst. Mater. 1994, 209, 870.

[49] K. Oh‐ishi , Y. Tsuchiya , Y. Iizuka , H. Yamane , J. Solid State Chem. 2001, 160, 251.

[50] M. Paranthaman , K. A. David , T. B. Lindemer , Mater. Res. Bull. 1997, 32, 165.

[51] F. C. M. Driessens , G. D. Rieck , H. N. Coenen , J. Inorganic Nuclear Chem. 1968, 30, 747.

[52] P. K. Davies , J. Electrochem. Soc. 1982, 129, 31C.

[53] K. Ruck , M. Wolf , M. Ruck , D. Eckert , G. Krabbes , K. H. Müller , Mater. Res. Bull. 2001, 36, 1995.

[54] K. Sparta , A. Löffert , C. Gross , W. Aßmus , G. Roth , Z. Kristallogr. – Cryst. Mater. 2006, 221, 782.

[55] M. Biesuz , J. Chen , M. Bortolotti , G. Speranza , V. Esposito , V. M. Sglavo , J. Mater. Chem. A 2022, 10, 23603.

[56] J. Deng , C. Gu , H. Xu , P. Zhu , G. Xiao , Adv. Funct. Mater. 2024, 34, 2315529.

[57] G. Karunakaran , M. Kundu , S. Kumari , E. Kolesnikov , M. V. Gorshenkov , G. Maduraiveeran , M. Sasidharan , D. Kuznetsov , J. Alloys Compd. 2018, 763, 94.

[58] U. Dahlborg , J. Cornide , M. Calvo‐Dahlborg , T. C. Hansen , A. Fitch , Z. Leong , S. Chambreland , R. Goodall , J. Alloys Compd. 2016, 681, 330.

[59] J. Fiocchi , A. Mostaed , M. Coduri , A. Tuissi , R. Casati , J. Alloys Compd. 2022, 910, 164810.

[60] C. Dejoie , M. Coduri , S. Petitdemange , C. Giacobbe , E. Covacci , O. Grimaldi , P.‐O. Autran , M. W. Mogodi , D. Šišak Jung , A. N. Fitch , J. Appl. Crystallogr. 2018, 51, 1721.

[61] M. Leoni , urn:isbn:978‐1‐118‐41628‐0 2019, 524.

[62] A. I. Ustinov , L. O. Olikhovska , N. M. Budarina , F. Bernard , in Diffraction Analysis of the Microstructure of Materials, (Eds: E. J. Mittemeijer , P. Scardi ), Springer, Berlin, Heidelberg 2004, pp. 333–359.

[63] M. Scavini , M. Coduri , M. Allieta , P. Masala , S. Cappelli , C. Oliva , M. Brunelli , F. Orsini , C. Ferrero , IUCrJ 2015, 2, 511. 10.1107/S2052252515011641 PMC4547819 26306193

[64] N. J. Usharani , A. Bhandarkar , S. Subramanian , S. S. Bhattacharya , Acta Mater. 2020, 200, 526.

[65] M. Coduri , M. Scavini , M. Allieta , M. Brunelli , C. Ferrero , J. Phys.: Conf. Ser. 2012, 340, 012056.

[66] D. N. Argyriou , J. Appl. Cryst. 1994, 27, 155.

[67] S. C. Tarantino , M. Giannini , M. A. Carpenter , M. Zema , IUCrJ. 2016, 3, 354. 28461896 10.1107/S2052252516012574 PMC5391857

[68] J. Conradie , Inorg. Chim. Acta 2019, 486, 193.

[69] A. Fitch , C. Dejoie , E. Covacci , G. Confalonieri , O. Grendal , L. Claustre , P. Guillou , J. Kieffer , W. de Nolf , S. Petitdemange , M. Ruat , Y. Watier , J. Synchrotron. Radiat. 2023, 30, 1003. 37462688 10.1107/S1600577523004915 PMC10481261

[70] G. B. Vaughan , R. Baker , R. Barret , J. Bonnefoy , T. Buslaps , S. Checchia , D. Duran , F. Fihman , P. Got , J. Kieffer , J. Synchrotron Radiat. 2020, 27, 515. 32153293 10.1107/S1600577519016813 PMC7842212

[71] B. H. Toby , R. B. Von Dreele , J. Appl. Cryst. 2013, 46, 544.

[72] T. Roisnel , J. Rodríquez‐Carvajal , Mater. Sci. Forum 2001, 378, 118.

