# Supplementary material for: Phase Stability of High Entropy (Mg,Ni,Co,Cu,Zn)O from Temperature‐Resolved Synchrotron Diffraction: Tetragonal Distortion and Guggenite Phase
Source: Small. 2024 Dec 19;21(5):2406634. doi: 10.1002/smll.202406634 (PMC11798356; doi:10.1002/smll.202406634)
Supplement: Supplementary file 1 — Supporting Information [file SMLL-21-2406634-s001.docx]

Supporting Information

Phase stability of high Entropy (Mg,Ni,Co,Cu,Zn)O from Temperature-resolved Synchrotron Diffraction: Tetragonal Distortion and Guggenite Phase

Mauro Coduri^1,2,^*, Martina Fracchia^1,2^, Stefano Checchia^3^, Maela Manzoli^4,2^, Catherine Dejoie^3^, Paolo Ghigna^1,2^, Umberto Anselmi-Tamburini^1,2^

**Table S1**. Impurities quantified by high resolution powder diffraction. Spinel and guggenite have to be intended as crystallographic phases rather than stoichiometric Co_3_O_4_ and Cu_2_MgO_3_. Indeed, a spinel phase is observed even in the sample without Co while guggenite occurred in the absence of Mg, thus li

|  | HEO | noCo | noZn | noCu | noMg | noNi |
| --- | --- | --- | --- | --- | --- | --- |
| spinel | 0.7 % | 0.7 % | - | 0.3 % | - | 0.5 % |
| guggenite | - | 0.7 % | - | - | 1.3 % | - |
| Pt | - |  | 0.01 % | - | - | - |


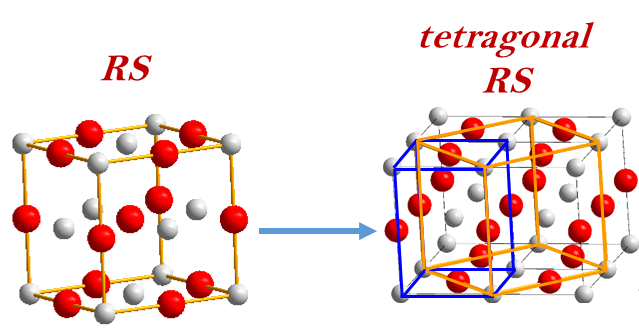


**Figure S1**. Sketch of the cubic to tetragonal phase transformation. Left: original cubic RS phase is depicted on the left. Grey balls stand for cations, red for oxygen. Right: the tetragonal unit cell is displayed in blue, while the original cubic unit cell is displayed in orange. It turns out that the cell parameter a_T_ is half of the face diagonal of the original cube, while c_T_ is the same as the cubic lattice parameter. The cations still lie in the origin, while the O is local in the middle of an edge.

**Figure S2.** a, c) HR-TEM representative images of the *HEO-5* sample. b) EDS maps of the region shown in panel a for all the cations and oxygen. d) Fast Fourier Transform of the image in c. Instrumental magnification: 400000×.

**Figure S3.** a) HR-TEM representative images of *noNi*. b) Fast Fourier Transform of the image in a. c) EDS maps of the regions shown in a for Mg, Co, Cu, Zn and oxygen. Instrumental magnification: 400000x.

**Figure S4.** a) HR-TEM representative images of *noCo*. b) Fast Fourier Transform of the image in a. c) EDS maps of the regions shown in a for Zn, Ni, Cu, O and Mg. Instrumental magnification: 400000x.

**Figure S5.** a) HR-TEM representative images of *noCu*. b) Fast Fourier Transform of the image in a. c) EDS maps of the regions shown in a for Mg, Ni, Co, Zn and oxygen. Instrumental magnification: 400000x.

**Figure S6.** a) HR-TEM representative images of *noZn*. b) Fast Fourier Transform of the image in a. c) EDS maps of the regions shown in a for Co, Ni, Cu, Mg and oxygen. Instrumental magnification: 400000x.


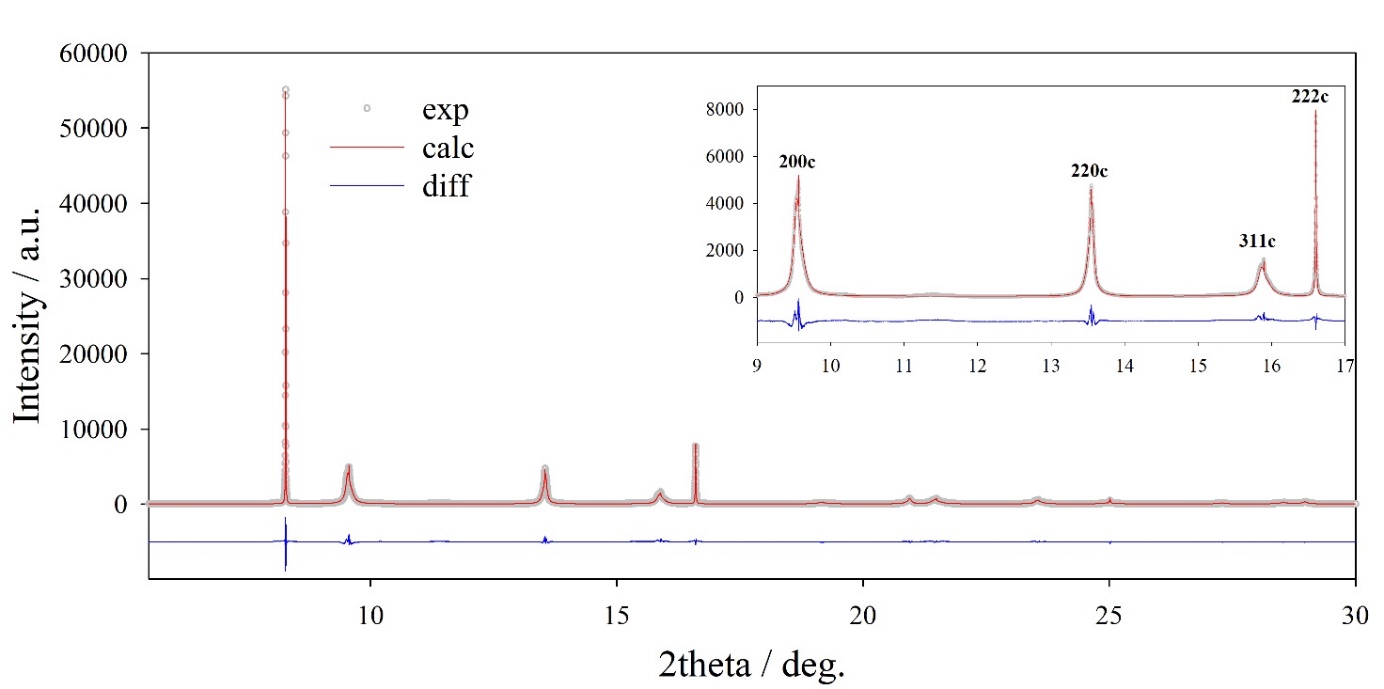


**Figure S7.** Rietveld refinement of *noMg* at RT against high resolution powder diffraction data. The Miller indexes in the inset refer to the cubic setting of the RS phase.


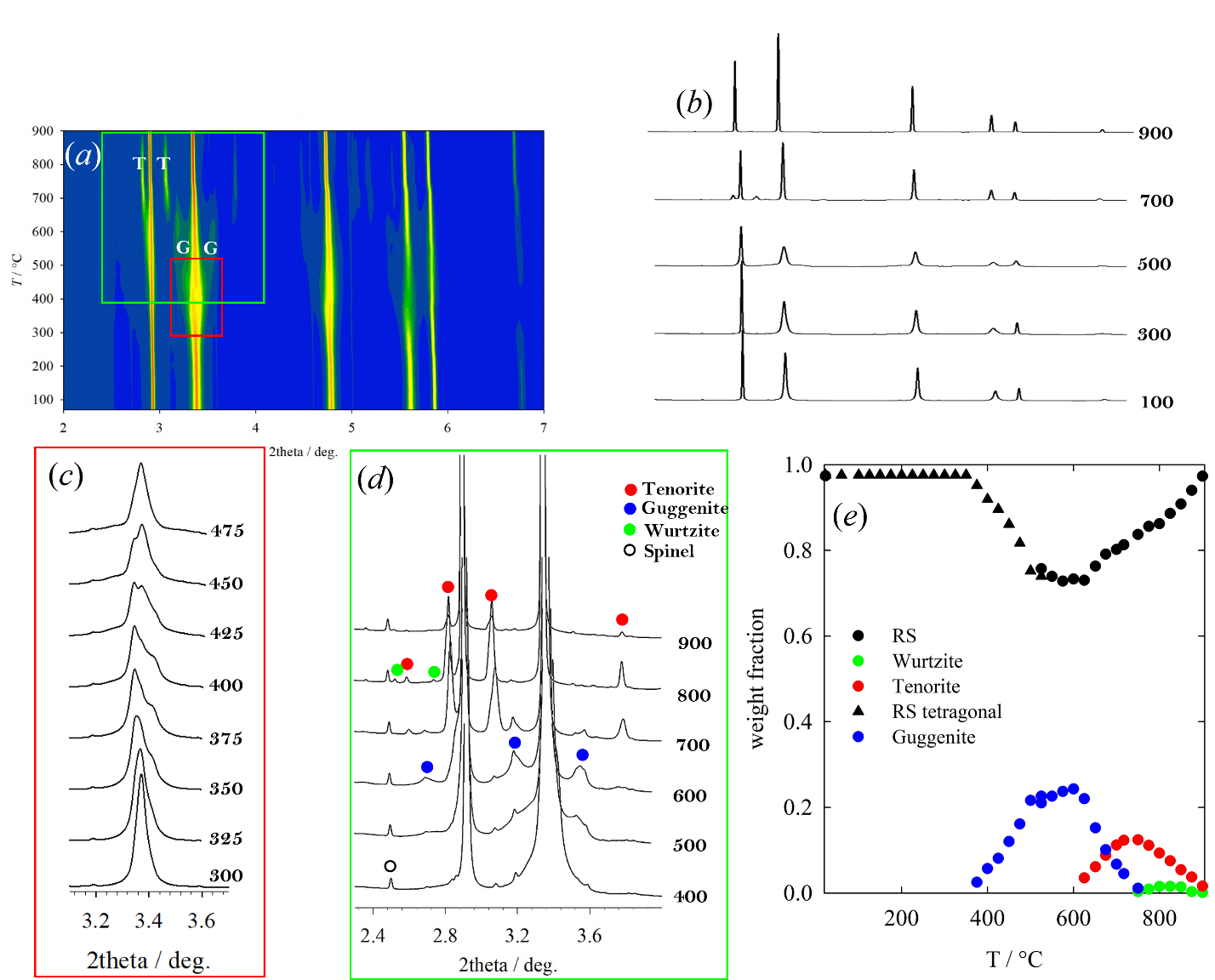


**Figure S8**. (*a*) Temperature-resolved XRD patterns of *noCo* reported in Log scale; T stands for tenorite, G for guggenite. (*b*) Full scale patterns in linear scale at some selected temperatures, indicated on the right-hand side. (*c*) Temperature evolution of the 200 reflection in RS setting from 300 to 475 °C, consistent with the red area in panel (*a*). (*d*) Low intensity scale highlighting the formation and evolution of secondary phases from 400 to 900 °C. (*e*) Weighted phase fractions computed by Rietveld refinements.


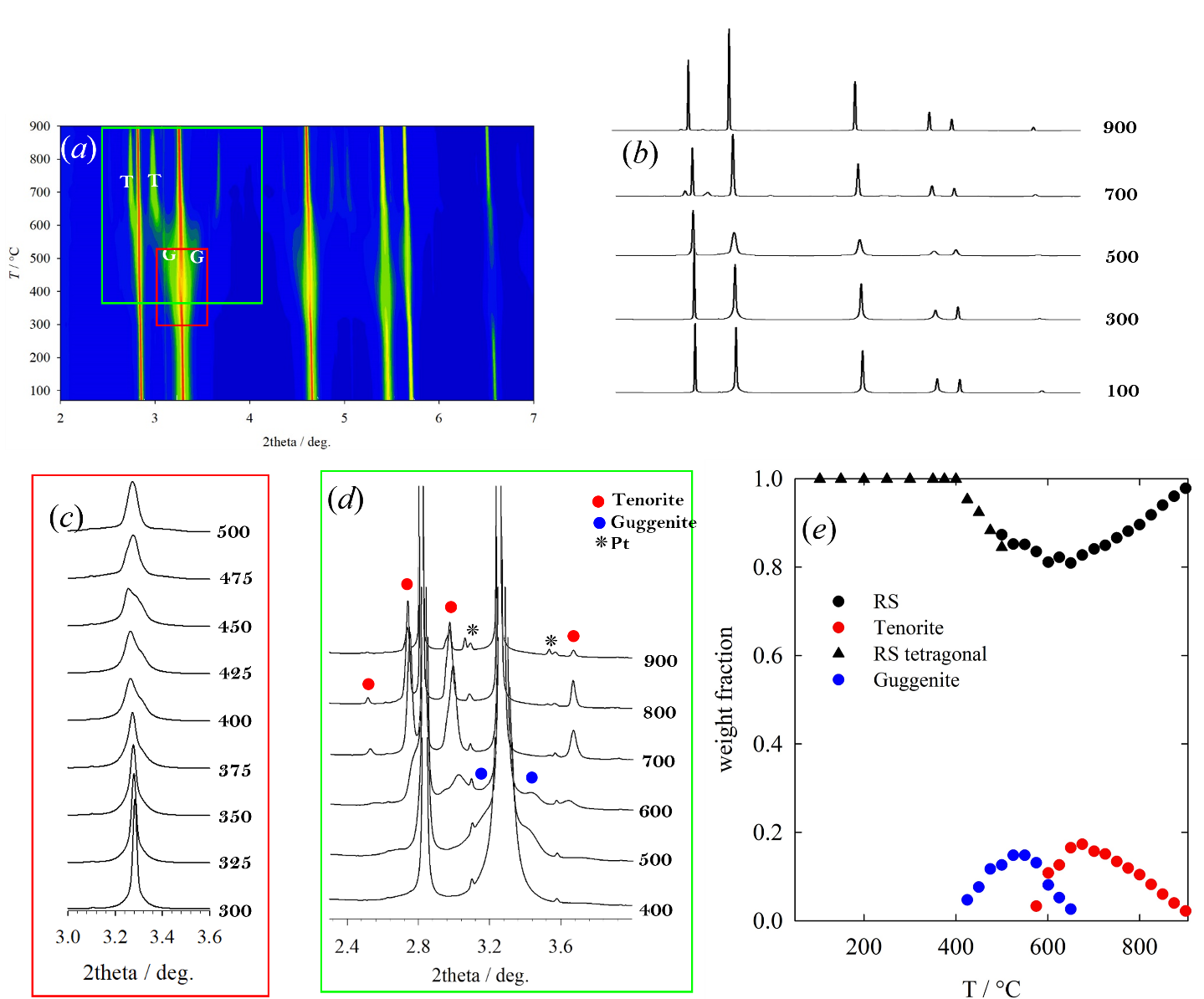


**Figure S9**. (*a*) Temperature-resolved XRD patterns of noZn reported in Log scale; T stands for tenorite, G for guggenite. (*b*) Full scale patterns in linear scale at some selected temperatures, indicated on the right-hand side. (*c*) Temperature evolution of the 200 reflection in RS setting from 300 to 500 °C, consistent with the red area in panel (*a*). (*d*) Low intensity scale highlighting the formation and evolution of secondary phases from 400 to 900 °C. (*e*) Weighted phase fractions computed by Rietveld refinements.


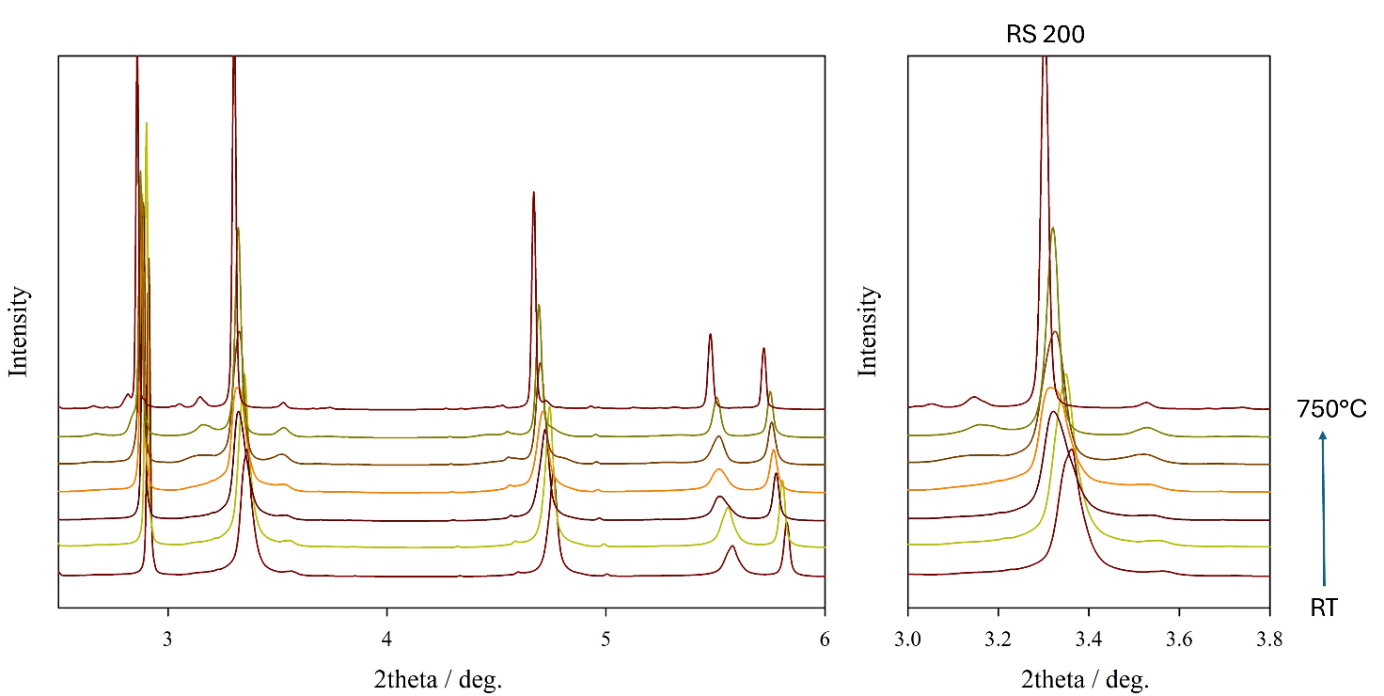


**Figure S10**. XRD pattern of *noNi* collected at some temperatures while heating from RT to 750°C. The same scheme observed for *noCo* and *noZn* is confirmed, i.e. a tetragonal distortion, followed by the formation of guggenite and, at higher temperature, tenorite.


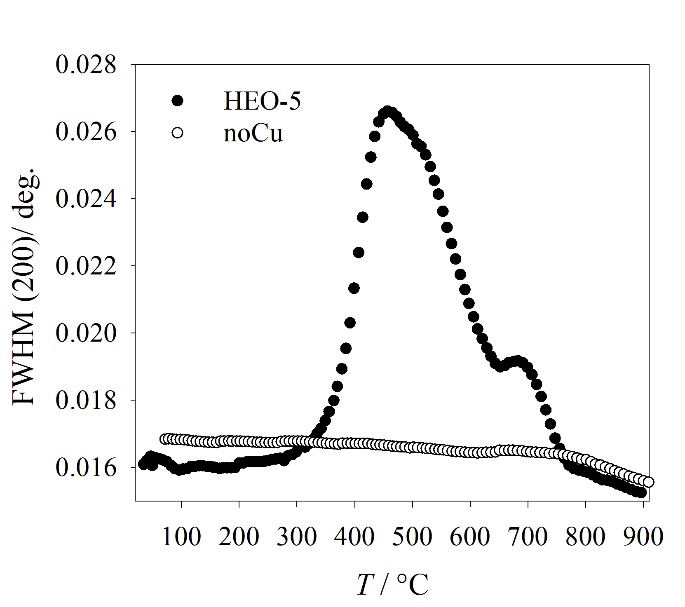


**Figure S11**. FWHM of the 200 reflection of the RS phase for *HEO-5* (full circles) and *noCu* (empty circles) specimens.


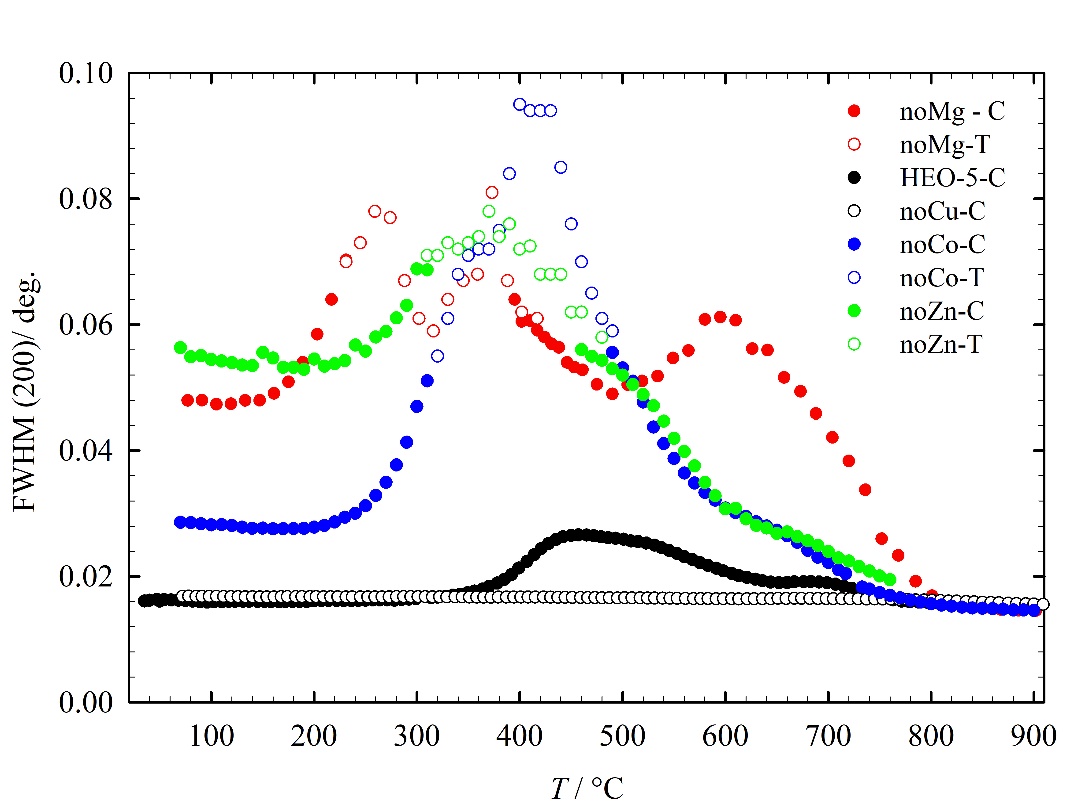


**Figure S12**. FWHM of the 200 reflection of the RS phase, either cubic (C, full symbols) or tetragonal (T, empty symbols) as a function of temperature.

| 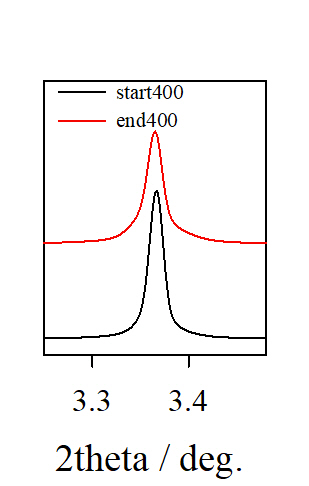 | 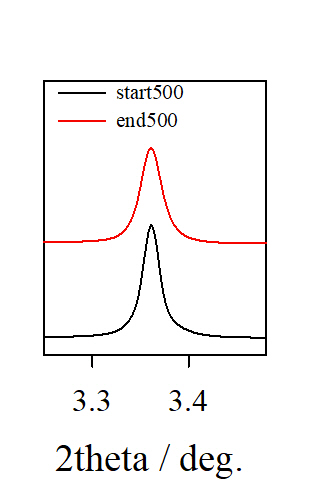 | 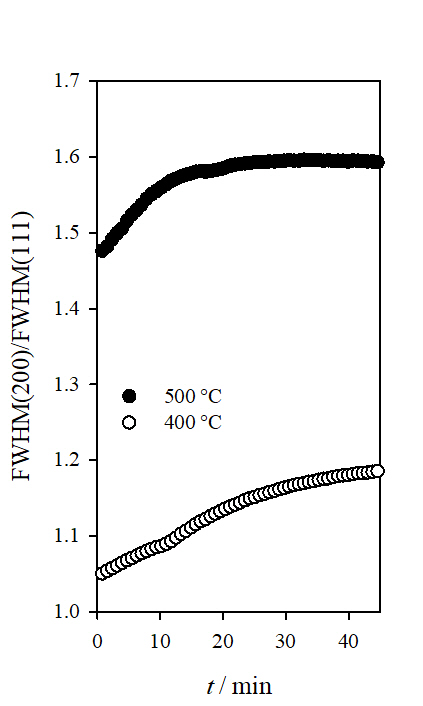 |
| --- | --- | --- |

**Figure S13**. XRD patterns at the beginning and the end of the thermalization at 400 and 500°C. The right-hand side plot shows the evolution of the ratio of the FWHM of 200 and 111 reflections. Whereas the 200 peak becomes broader during the isotherm, especially at 500°C, no peak splitting nor asymmetry, evidence of long range tetragonal distortion, are observed.


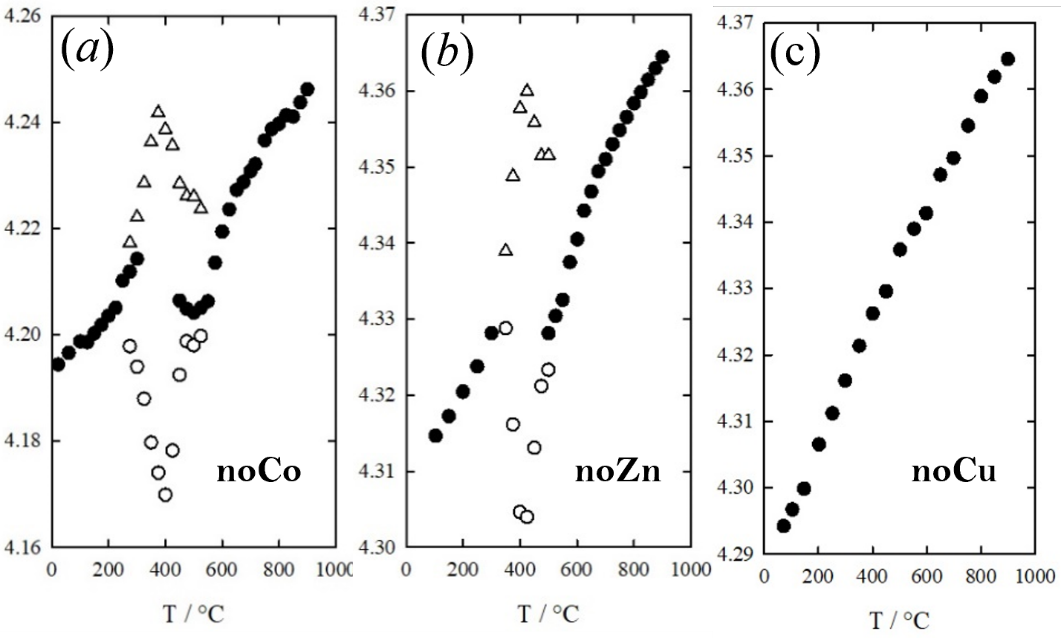


**Figure S14.** Evolution of lattice parameter of the cubic and tetragonal RS phase for *noCo*, *noZn* and *noCu*. Full circles refer to the cubic RS phase, empty triangles to the tetragonal phase, with a normalized to the cubic setting.


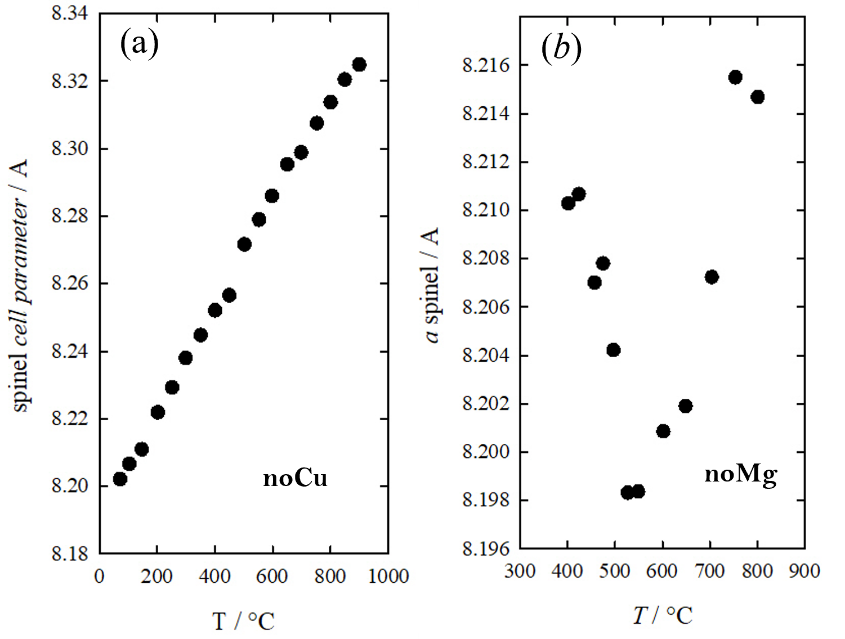


**Figure S15.** Evolution of lattice parameter of the spinel phase observed in *noCo* and *noMg*.


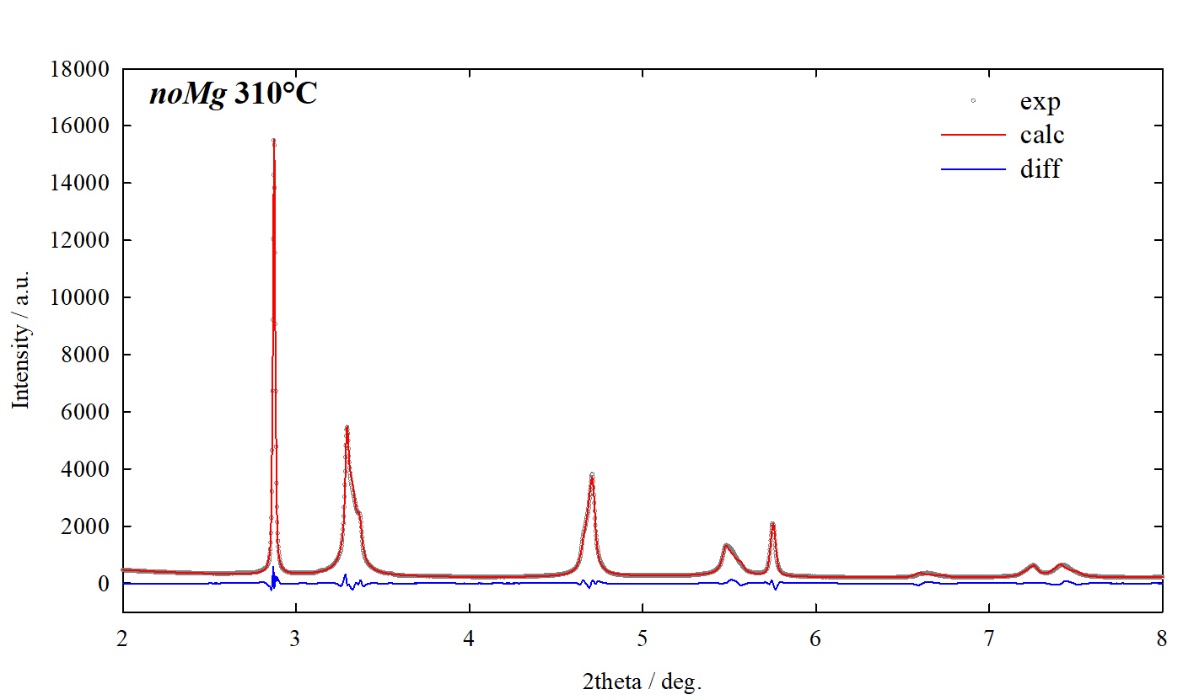


**Figure S16.** Rietveld refinement of *noMg* at 310°C against powder diffraction data. Grey points: experimental, red solid line: calculated, blue solid line: difference.

**Table S2.** Ionic radii for the elements of interest of this study taken from[1] in tetrahedral (IV) and octahedral (VI) coordination. When multiple electronic configurations are available, the high spin one is considered.

|  | +2 | | +3 |
| --- | --- | --- | --- |
|  | **IV** | **VI** | **VI** |
| Ni | 0.55 | 0.69 | 0.60 |
| Mg | 0.57 | 0.72 | - |
| Cu | 0.57 | 0.73 | - |
| Zn | 0.60 | 0.74 | - |
| Co | 0.58 | 0.745 | 0.61 |

**TextS1. CuO-MgO.** In order to understand the growing behavior of guggenite, we investigated the microstructural relationships between RS, guggenite and tenorite. Considering that guggenite is reported as a stable phase within the CuO-MgO phase diagram,[2] we produced a specimen with composition Mg_0.8_Cu_0.2_O, that annealed at 950 °C showed the coexistence of tenorite and guggenite secondary phases. The SEM images using BSE reported in Figure S17 revealed the presence of a Mg-rich RS matrix (83% at. Mg, 17% Cu), into which the secondary phases are embedded forming Cu-rich precipitates. The inner part is composed of CuO, while the intermediate part has mixed Cu-Mg cation composition (73% Cu, 27% Mg). This represents the composition of guggenite, which in the case of Mg has been reported to be stabilized by an excess of Cu[3].

The SEM images were collected on a SEM TESCAN Mira 3 XMU instrument. A fraction of each pellet was embedded into an epoxy resin. Once hardened, they were polished from SiC abrasive coarse papers to finer diamond clothes and using colloidal silica. Backscattered electrons (BE) were employed to check for sample homogeneity on the micrometric scale.


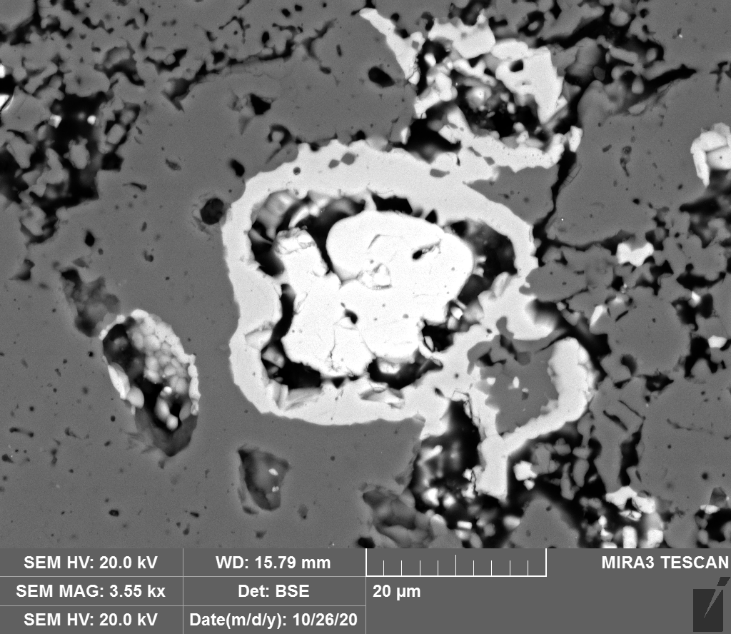


**Figure S17.** SEM-BSE image of a Mg_0.8_Cu_0.2_O specimen treated at 950°C.


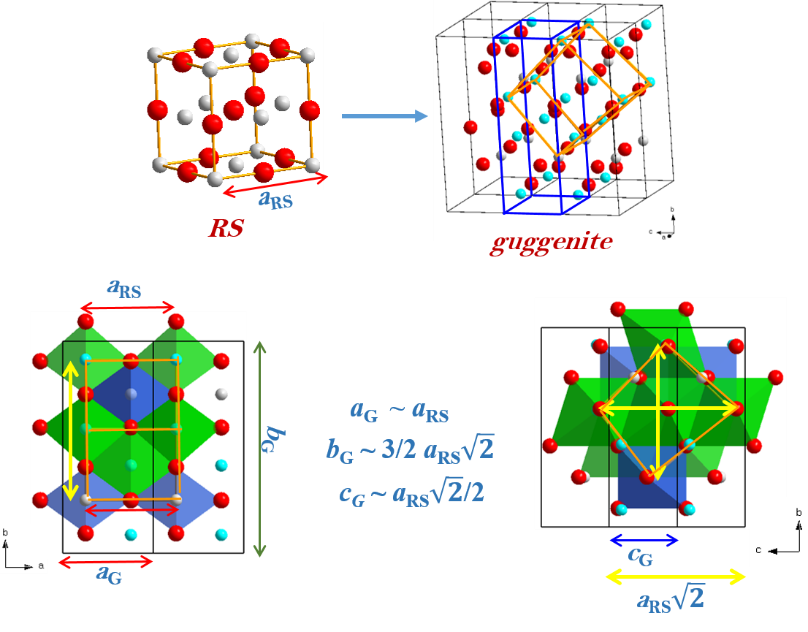


**Figure S18**. Structural relationships between RS and guggenite. On the top of the figure the original RS unit phase is highlighted as orange lines while the guggenite unit cell is in blue lines. Red balls: O ions, grey balls and blue octahedra, general bivalent cation, cyan balls and green octahedra: Cu^2+^ ions. The bottom part describes the guggenite cell parameters (*a*_G_, *b*_G_ and *c*_G_) with respect to that of RS (*a*_RS_).

**TextS2.**

The chemical composition of the phases of interest in the (Mg, Ni, Co, Zn, Cu)O system are discussed. Concerning rock salt, CoO, NiO and MgO are known to have full reciprocal solubility to form a solid solution; Zn^2+^ at high temperature has large solubility into NiO, CoO and MgO, [4–6] while Cu has been reported to dissolve up to at least χ(Cu)=0.20 into each of the RS oxides at ~1000°C. [2,7–10] Therefore, from the sole presence of the RS signals it is not possible to derive the elemental composition. Similarly, the presence of a spinel phase does not imply the observation of simple Co_3_O_4_ and indeed, cobaltites of Ni, Zn, Mg, and Cu have been reported[11–13]. Solubility of divalent cations into tenorite is very limited (*e.g.* ~4% of Co up to ~1020°C[7,10]), therefore the presence of tenorite can be taken as a signature of actual CuO. Co was reported to dope wurtzite up to ~17% at 1000°C[6], while solubility of the other cations has been reported only to a few percent units[6,14]. Tenorite and wurtzite are not miscible.[15] Again, we assume that the formation of wurtzite upon heating is related to pure ZnO.

The composition of the spinel phase observed upon heating can be discussed in view of the above comments. In the *noMg* specimen, as neither tenorite nor guggenite is observed up to 550°C; the phase most likely to host Cu is the spinel. In fact, the restoration of the cubic RS phase is accompanied by an important enrichment in spinel, which is estimated to reach ~40% at 625°C. The spinel unit cell contracts progressively from its nucleation at ~400 °C (8.210 Å) to ~550°C (8.198 Å). This suggests a change in composition. In fact, the spinel secondary phase that forms in *noCu*, likely Co_3_O_4_, shows linear thermal expansion. (Fig. S10) This discrepancy suggests that the newly formed spinel phase gradually takes up some Cu and possibly Ni and/or Zn.

**References:**

[1] R. D. Shannon, *Acta Cryst A* **1976**, *32*, 751.

[2] M. Paranthaman, K. A. David, T. B. Lifidemer’, **n.d.**, *32*, 9.

[3] *Zeitschrift für Kristallographie - Crystalline Materials* **1994**, *209*, 870.

[4] S. Raghavan, J. P. Hajra, G. N. K. Iyengar, K. P. Abraham, *Thermochimica Acta* **1991**, *189*, 151.

[5] “X-Ray Diffraction Studies of the Solid State Reaction in the NiO-ZnO System - Cerca con Google,” can be found under https://www.google.com/search?channel=nrow5&client=firefox-b-d&q=X-Ray+Diffraction+Studies+of+the+Solid+State+Reaction+in+the+NiO-ZnO+System, **n.d.**

[6] A. Navrotsky, A. Muan, *Journal of Inorganic and Nuclear Chemistry* **1971**, *33*, 35.

[7] F. C. M. Driessens, G. D. Rieck, H. N. Coenen, *Journal of Inorganic and Nuclear Chemistry* **1968**, *30*, 747.

[8] J. Bularzik, P. k. Davies, A. Navrotsky, *Journal of the American Ceramic Society* **1986**, *69*, 453.

[9] N. G. Schmahl, J. Barthel, G. F. Eikerling, *Zeitschrift für anorganische und allgemeine Chemie* **1964**, *332*, 230.

[10] L. A. Zabdyr, O. B. Fabrichnaya, *Calphad* **2004**, *28*, 293.

[11] K. Krezhov, P. Konstantinov, *Physica B: Condensed Matter* **1997**, *234–236*, 157.

[12] D. Klissurski, E. Uzunova, *J Mater Sci* **1994**, *29*, 285.

[13] K. Petrov, K. Krezhov, P. Konstantinov, *Journal of Physics and Chemistry of Solids* **1989**, *50*, 577.

[14] C. H. Bates, W. B. White, R. Roy, *Journal of Inorganic and Nuclear Chemistry* **1966**, *28*, 397.

[15] L. Xia, Z. Liu, P. A. Taskinen, *Ceramics International* **2016**, *42*, 5418.
